# Key role of TLR3 in type I IFN expression and apoptosis induction in IBDV-infected chicken fibroblast cells

**DOI:** 10.3389/fcimb.2026.1767950

**Published:** 2026-03-06

**Authors:** Elisabet Diaz-Beneitez, Leyre Concostrina-Martínez, Liliana L. Cubas-Gaona, Altea Martín-Martínez, Juan R. Rodríguez, José F. Rodríguez, Fernando Almazán, Dolores Rodríguez

**Affiliations:** Department of Molecular and Cellular Biology, Centro Nacional de Biotecnología (CNB), Consejo Superior de Investigaciones Científicas (CSIC), Madrid, Spain

**Keywords:** IBDV, apoptosis, innate immune response, interferon, TLR3, RIPK1

## Abstract

Infectious Bursal Disease Virus (IBDV) (*Avibirnavirus* genus, *Birnaviridae* family) is a non-enveloped virus with a double-stranded RNA (dsRNA) genome. IBDV causes a highly contagious and immunosuppressive disease in domestic chickens (*Gallus gallus*), representing a major threat to the global poultry industry. Apoptotic cell death and exacerbated innate immune responses have been implicated in IBDV pathogenesis. Previous studies from our laboratory demonstrated the crucial role of type I interferon (IFN) in triggering apoptosis in IBDV-infected cell cultures. Genomic IBDV dsRNA is recognized by the cytoplasmic pattern recognition receptor (PRR) melanoma differentiation-associated gene 5 (MDA5) in chicken cells, triggering type I IFN responses. However, the contribution of the endosomal PRR Toll-like receptor 3 (TLR3) dsRNA sensor on type I IFN production upon IBDV infection has not been studied, despite several studies have demonstrated that its expression is significantly upregulated upon IBDV infection. Here, we demonstrate that ablation of TLR3 gene expression in DF-1 chicken fibroblasts results in a complete blockade of IBDV-induced apoptosis, a marked reduction in IFN production, and a significant enhancement of virus progeny yields. Notably, this effect appears to be specific to IBDV, as it was not observed with any of the other RNA viruses tested, including single-stranded RNA (ssRNA) viruses such as vesicular stomatitis virus (VSV), Semliki Forest virus (SFV), and Newcastle disease virus (NDV), nor even with the dsRNA virus avian reovirus (ARV). Our findings also suggest that TLR3 may also play a role in viral release into the extracellular space. Additionally, receptor interacting protein kinase 1 (RIPK1), a protein that interacts with TLR3 through the adaptor Toll/IL-1 receptor (TIR) domain-containing adaptor-inducing interferon-β (TRIF), was shown to contribute to both IFN production and apoptosis in response to IBDV infection or dsRNA stimulation in DF-1 cells. Overall, this study provides new insights into the innate immune recognition of IBDV, highlighting the central role of TLR3 in mediating antiviral responses in chicken cells.

## Introduction

Infectious Bursal Disease Virus (IBDV), a member of the family *Birnaviridae* and the sole representative of the genus *Avibirnavirus*, is a non-enveloped virus with a bipartite double-stranded RNA (dsRNA) genome. IBDV causes a highly contagious and devastating immunosuppressive disease in young chickens (*Gallus gallus*) between 3 and 6 weeks of age. The main target cells of IBDV are immature B lymphocytes located in the bursa of Fabricius, the main lymphoid organ in birds. IBDV causes a massive depletion of infected pre-B lymphocytes and the atrophy of the bursa of Fabricius, resulting in a severe immunosuppression that predisposes chicks to secondary infections and limits the efficacy of vaccines against other important poultry pathogens. Currently, the prevalence of very virulent IBDV strains, along with the emergence of novel reassortant and recombinant strains in the field across several countries, poses a major threat to poultry industry and is responsible of significant economic losses worldwide (Wang et al., 2023; Abd El-Fatah et al., 2024; Legnardi et al., 2023). Therefore, a comprehensive understanding of the mechanisms underlying IBDV-host interactions is essential for the development of effective and innovative control strategies.

IBDV virions are naked icosahedral particles containing two segments of dsRNA of 3.2 and 2.8 kbp (segments A and B, respectively). It has been proposed that upon receptor recognition, IBDV virions enter the cell through a macropinocytosis mechanism and subsequently traffic through the endocytic pathway (Yip et al., 2012; Gimenez et al., 2015). During virus infection five mature viral proteins (VP1 to VP5) are synthesized. Four of these proteins are encoded by segment A, which carries two partially overlapping open reading frames (ORF). The first one (ORF A1) encodes the nonstructural protein VP5, which is dispensable for viral replication in cultured cells (Mundt et al., 1997) but is essential for cell-to-cell transmission *in vitro* (Méndez et al., 2015, 2017) and for viral pathogenesis *in vivo* (Yao et al., 1998). The second ORF (ORF A2) encodes a polyprotein precursor, which is self-cleaved co-translationally by the viral protease VP4 (Birghan et al., 2000), resulting in the formation of the VP2 precursor (pVP2), VP3 and VP4. pVP2 undergoes further cleavages to generate the mature VP2 capsid protein (Irigoyen et al., 2012). Proteolytic processing of pVP2 also produces four small amphipathic peptides that remain associated with the viral capsid and are believed to play a crucial role during the early stages of infection. One of these peptides, pep46, released during capsid disassembly (a process triggered by the low calcium concentration and acidic pH characteristic of mature endosomal compartments), is thought to induce pore formation in the endosomal membrane, enabling the release of the viral genome into the cytoplasm (Galloux et al., 2007). Segment B contains a single ORF that encodes VP1, a multifunctional polypeptide with an RNA-dependent RNA Polymerase (RdRp) activity (von Einem et al., 2004). Within the capsid, the viral genome is structured in ribonucleoprotein complexes (RNPs), where the dsRNA is coated by VP3 and complexed with VP1 bound to the 5’-end of the genome (Luque et al., 2009).

Although the molecular mechanisms underlying IBDV pathogenicity remain poorly understood, accumulating evidence indicates that the exacerbation of the innate immune response and the apoptosis of infected cells are key contributors to disease severity (Ingrao et al., 2013). Significantly, previous studies from our laboratory showed that exposure to type I interferon (IFN) of HeLa cells infected with IBDV triggers a rapid and massive apoptotic cell death response, suggesting that IFN may play a critical role in the pathogenesis associated with this virus (Cubas-Gaona et al., 2018).

Type I IFNs are central to the host defense against viral infections. Upon binding to their membrane-bound receptor complex, IFN-α/β receptor (IFNAR), they initiate the Janus kinase-signal transducer and activator of transcription protein (JAK/STAT) signaling pathway, leading to the transcription of a broad repertoire of IFN-stimulated genes (ISGs). These ISGs encode proteins with diverse functions, including antiviral, immunomodulatory, cell cycle-inhibitory, and pro-apoptotic activities. Among the best characterized pro-apoptotic ISGs is the dsRNA-activated protein kinase (PKR), which plays a key role in linking antiviral signaling to programmed cell death (Kaufman, 1999; McAllister and Samuel, 2009).

During viral infection, host cells rapidly recognize pathogen-associated molecular patterns (PAMPs), such as viral RNA or DNA, through pattern recognition receptors (PRRs), including retinoic acid-inducible gene I-like (RIG-I) receptors (RLRs) and Toll-like receptors (TLRs). Upon ligand binding, these sensors activate downstream adaptor proteins, such as the mitochondrial antiviral-signaling protein (MAVS) for RLRs, and the Toll/IL-1 receptor (TIR) domain-containing adaptor-inducing interferon-β (TRIF) or myeloid differentiation primary response 88 (MyD88) for TLRs. They initiate signaling cascades that converge on the activation of transcription factors such as IFN regulatory factors 3 and 7 (IRF3/7) and nuclear factor kappa-light-chain-enhancer of activated B cells (NF-κB), leading to the transcription of type I IFNs (IFN-α/β) and other immune effector genes (Akira et al., 2006). In chickens, which lack the *RIG-I* gene, cytoplasmic recognition of viral dsRNA relies on melanoma differentiation-associated gene 5 (MDA5), the only RLR of this class expressed in these birds. Previous studies have shown that IBDV genomic dsRNA is sensed by MDA5, which signals through MAVS to activate IRF7—the only IRF of this type present in chickens—and NF-kB, ultimately promoting the expression of type I IFN and other proinflammatory cytokines (Lee et al., 2014; Ye et al., 2014; Lee et al., 2015b; Diaz-Beneitez et al., 2022).

As mentioned above, PKR is an ISG, but it is also a sensor for dsRNA. Our earlier work revealed that PKR can also bind IBDV dsRNA in the cytoplasm of infected cells, leading to upregulation of IFN-β expression and acting as a key mediator of the apoptotic response in IBDV infected human HeLa cells (Cubas-Gaona et al., 2018). However, the role of PKR in the context of IBDV-infected chicken cells remains almost unexplored. Nonetheless, it has been shown that the IBDV-encoded VP3 protein is capable of antagonizing PKR-mediated apoptotic signaling, suggesting a potential viral strategy to modulate this pathway in chicken cells (Busnadiego et al., 2012).

Viral dsRNA can also be recognized by the membrane associated PRRs of the TLR family. In chickens, TLR3 and TLR7 are the only TLRs known to participate in the recognition of RNA viruses, detecting dsRNA and single-stranded RNA (ssRNA), respectively (Chen et al., 2013). Evidence from multiple studies shows that TLR3 expression increases during IBDV infection both *in vitro* and *in vivo*, supporting its contribution to the antiviral innate immune response (Kumar et al., 2010; Rauf et al., 2011; Guo et al., 2012; Smith et al., 2015; Lee et al., 2015a; He et al., 2017; Chen et al., 2022). However, a direct link between TLR3 and the establishment of antiviral response against IBDV has not been reported as yet.

TLRs are type I transmembrane proteins comprising three primary domains: (i) an extracellular N-terminal ligand binding domain; (ii) a single-pass transmembrane helix that mediates membrane anchoring; and (iii) a cytoplasmic TIR domain, which facilitates protein–protein interactions essential for downstream signal transduction (Akira et al., 2006). In mammalian cells, the interaction of TLR3 with dsRNA triggers a complex signaling pathway that begins with TLR3 dimerization, the phosphorylation of a tyrosine residue in the TIR domain, and the recruitment of the adaptor protein TRIF (Yamamoto et al., 2003). Subsequently, TRIF facilitates the engagement of tumor necrosis factor (TNF) receptor-associated factor 3 (TRAF3), which leads to the association of two kinases, TANK binding kinase 1 (TBK1) and IkappaB kinase-epsilon (IKKϵ) (Sato et al., 2003) through a signalosome complex, and the activation of IRF3/7. In parallel, the recruitment of TRAF6 and the receptor interacting protein kinase 1 (RIPK1) triggers the activation of NF-kB (Alexopoulou et al., 2001). In chicken cells, the signaling pathway downstream of TLR3 remains poorly characterized. Nonetheless, the identification of functional homologs for most key mammalian components, including TRIF, TBK1, IKKϵ, and RIPK1, in the chicken genome (*Gallus gallus*) suggests a conserved signaling mechanism across these species (Gillespie et al., 2011).

In this study, we investigated the molecular mechanisms underlying apoptosis induction in IBDV-infected chicken cells. Our findings reveal a strong link between IFN-mediated activation of the JAK/STAT signaling pathway and the initiation of apoptosis. We also examined the contribution of the cytoplasmic and endosomal dsRNA sensors—MDA5, PKR, and TLR3—to IFN expression and cell death. Our results demonstrate that the three PRRs participate in this process; however, TLR3 emerges as the primary mediator, as its ablation completely abrogates apoptosis in IBDV-infected cells. Furthermore, we have identified the downstream signaling molecule RIPK1 as a key effector in TLR3-mediated apoptotic signaling.

## Materials and methods

### Cells, viruses and infections

DF-1 (chicken embryonic fibroblasts, ATCC CRL-12203), CEF (primary chicken embryo fibroblasts, Charles River Laboratories, kindly provided by Dr. Mariano Esteban´s lab at Centro Nacional de Biotecnología, CSIC, Madrid, Spain), QM7 (quail muscle myoblasts, ATCC CRL-1962) and DT40 cells (immortalized chicken B cell line infected persistently with avian leukosis virus (ALV)) (Baba et al., 1985) cells were grown in Dulbecco´s modified minimal essential medium (DMEM) supplemented with penicillin (100 U/ml), streptomycin (100 U/ml), gentamicin (50 µg/ml), fungizone (12 µg/ml), nonessential amino acids, and 10% fetal calf serum (FCS; Sigma-Aldrich).

IBDV infections were performed on preconfluent (70-80%) cell monolayers with the Soroa strain, a cell-adapted, serotype 1 virus (Lombardo et al., 1999), diluted in DMEM at a multiplicity of infection (MOI) of 2 plaque forming units (PFU) per cell (PFU/cell), unless otherwise stated. After 1 h of adsorption at 37°C, the medium was removed and replaced with fresh DMEM supplemented with 2% FCS. Infected cells were incubated at 37°C until the specified times post-infection (pi).

Infections with vesicular stomatitis virus (VSV) expressing GFP (VSV-GFP) (Ostertag et al., 2007), avian reovirus (ARV) strain S1133 (Kindly provided by Dr. José Manuel Martínez Costas, CIQUS, University of Santiago de Compostela, Spain), Semliki Forest virus (SFV) (Kindly provided by Dr. Christian Smerdou, CIMA, Universidad de Navarra, Pamplona) were performed as described above on preconfluent (70-80%) DF-1 cell monolayers at an MOI of 2 PFU/cell. Infections with Newcastle disease virus (NDV) expressing GFP (NDV-GFP) (Kindly provided by Dr. Adolfo García-Sastre, Icahn School of Medicine at Mount Sinai, New York, NY, United States) were performed at MOIs of 0.1 and 1PFU/cell.

### Antibodies and reagents

The following antibodies were used in Western blot: anti-β-actin mAb (Santa Cruz Biotechnology, #sc-47,778), rabbit polyclonal serum against the chicken PKR protein (kindly provided by Dr. Javier Benavente, University of Santiago de Compostela, Spain), and a polyclonal serum against the IBDV VP3 protein (Fernandez-Arias et al., 1998).

The expression, purification, and titration of recombinant chicken IFN-α (chIFN-α) were previously described (Cubas-Gaona et al., 2018). Bafilomycin A1 (BFA) and Staurosporine were purchased from Sigma-Aldrich, Poly I:C (high molecular weight) from InvivoGen, z-VAD-fmk (pan-caspase inhibitor) from Calbiochem, 7-deaza-2´-C-metiladenosina (7DMA) from Santa Cruz Biotechnology and Ruxolitinib (Rx) from Selleckechem.

### Plasmids

The *chTLR3* gene fused to a His-tag sequence at the 3´end was chemically synthesized (GenScript) and cloned into the pcDNA3 vector (pc-chTLR3-His). The IFN-β reporter plasmid (pLUCTER) (King and Goodbourn, 1994) was kindly provided by Dr. Stephen Goodbourn (St. George’s University of London, London, United Kingdom). The *Renilla* luciferase plasmid (pRL-null; Promega) was kindly provided for Dr. Pablo Gastaminza (Centro Nacional de Biotecnología, CSIC, Madrid, Spain). The NF-κB reporter plasmid (pSI-chNFκB-Luc) was generated as previously described (Diaz-Beneitez et al., 2022).

### Virus titration

For extracellular virus titrations, media from infected cell cultures were collected at 16 and 24 h pi and were subjected to low-speed centrifugation (2,000xg for 5 min) to remove cell debris. The resulting supernatants were used to determine virus titers. For intracellular virus titrations, cell monolayers were harvested by gentle scrapping, followed by low-speed centrifugation and pellets were suspended in fresh medium and subjected to three freeze and thaw cycles. Samples were subsequently centrifuged (2,000xg for 5 min) to dislodging virus particles from cell debris, and then used to determine intracellular virus titers. Virus titers were determined by plaque assay in QM7 cells using semisolid agar overlays followed by immunostaining as previously described (Méndez et al., 2015). For infections with NDV-GFP viral replication was estimated by measuring GFP expression. For this, fluorescence was quantified using a SpectraMax iD3 fluorometer (Molecular Devices). Cells were harvested as described above and cell pellets were resuspended in phosphate-buffered saline (PBS) and transferred to a black, clear-bottom 96-well microplate (Costar). Fluorescence was subsequently measured at an excitation wavelength of 395 nm and an emission wavelength of 509 nm.

### Luciferase reporter assays

Preconfluent (70-80% confluence) DF-1 cell monolayers were transfected in 24-well plates with either 100 ng of pLUCTER together with 30 ng of the pRL-null to normalize for transfection efficiency, or with 50ng of pSI-chNFκB-Luc, in combination with the pcDNA3-chTLR3-His or the empty pcDNA3 as control, as indicated in the results section, using Lipofectamine 2000 (Invitrogen) at a 1:2 ratio in Opti-MEM (Gibco) medium. 8 h later, cells were transfected with 250 ng of Poly I:C. After 16 h, cells were lysed, and luciferase assays were performed with the dual-luciferase assay kit (Promega) according to the manufacturer´s instructions. Luciferase activity was recorded using an Appliskan luminometer (Thermo Scientific). Firefly luciferase values were normalized to *Renilla* values, and the fold induction was calculated as the ratio of samples transfected with Poly I:C versus mock-transfected samples.

### Western blot assay

Infected cells were lysed in Laemmli´s sample buffer (62.5 mM Tris-HCL [pH 6.8]), 2% sodium dodecyl sulfate [SDS], 0.01% bromophenol blue, 10% glycerol, and 5% β-mercaptoethanol). Protein samples were subjected to 10% SDS-Polyacrylamide gel electrophoresis (PAGE), followed by electroblotting onto nitrocellulose membranes (Bio-Rad). Membranes were incubated with blocking buffer (Tris-buffered saline [TBS] containing 0.05% Tween 20 [TBST] and 5% nonfat dry milk) for 30 min at room temperature, and incubated at 4°C overnight with the corresponding primary antibodies diluted in blocking buffer. Thereafter, membranes were washed with TBST and incubated with either a goat anti-rabbit IgG-peroxidase (Sigma) or a goat anti-mouse IgG-peroxidase conjugate (Sigma), and immunoreactive bands were detected by an enhanced chemiluminescence (ECL) reaction (SuperSignal Thermo Scientific) and recorded using the ChemicDoc Touch Imaging System (Bio-Rad).

### Caspase 3/7 activity assays

Quantification of caspase activity was carried out by using the Caspase-Glo 3/7 kit (Promega). For this, DF-1 cell monolayers grown in 24-well plates (70-80% confluence) were infected in duplicate. At 24 h pi, cells were harvested in medium and kept frozen until their analysis. To that, 25 µl of the cell lysates under study was added to the same volume of Caspase-Glo 3/7 reagent in a 96-well plate. Plates were gently shaken and then incubated in the dark at room temperature for 1 h. The luciferase activity was recorded using an Appliskan luminometer (Thermo Scientific).

### Genome editing with CRISPR/Cas9

We used the Alt-R CRISPR-Cas9 system developed by Integrated DNA Technologies (IDT) to delete the two N-terminal dsRNA binding domains and a portion of the active site of the protein of PKR. Two sequence-specific CRISPR RNAs (crRNAs) for the *PKR* gene were designed using the Breaking-Cas website (https://bioinfogp.cnb.csic.es/tools/breakingcas/). The selected crRNA sequences were 1: 5’-CAAGCTCGATTACGTCGACGG-3’ and 2: 5’-TAGTGCGATATTATTGCAGCG-3’. These crRNAs, the conserved transactivating crRNA (tracrRNA) required for the formation of the guide RNA (gRNA) and the Cas9 nuclease were purchased from IDT. Following the manufacturer´s protocol, tracrRNA and crRNA were combined, and the resulting crRNA-tracrRNA duplexes were then mixed with the Cas9 to obtain ribonucleoprotein complexes (RNPs) that were transfected into the DF-1 cells plated in 6-well plates using Lipofectamine RNAiMAX (Invitrogen) in Opti-MEM medium according to the manufacturer´s instructions, and then seeded in 6-well plates. At 48 h post-transfection (pt), DF-1 cells monolayers were tripsinized, diluted and seeded in 100 mm dishes to obtain isolated clones. Individual clones were selected and grown, and subsequently analyzed by amplifying the regions of genomic DNA around the site of interest with the following primers: F1: 5´- GTGCATGGCCAAGATCAACAG-3´; R1: 5´-TCACGAGACCAAAGTCACCAA-3´ and R2: 5´- TGAGGAGGGCTAGTAAAACAGTAA-3´, used in two combinations, F1-R1 and F1-R2. Also, we analyzed the expression of PKR protein through Western blot. The results confirmed the mutation introduced in the *PKR* gene and the abrogation of PKR expression.

In addition, we also generate DF-1 TLR3 KO cell lines. We used the same system to partially delete the extracellular domain of chicken TLR3. Three sequence-specific crRNAs for the *chTLR3* gene were designed using the Breaking-Cas website. The selected crRNA sequences were 1: 5’-AACATGTTAGTGGTAATCCGTGG-3’; 2: 5’-GCCTAAATATCACGGTACTCTGG-3’ and 3: 5’-CAATTGCACGAACTC-CCTGATGG-3’. The following two pairs of crRNAs were used to generate different deletions in *TLR3* gene: crRNA 1 combined with crRNA 2, or crRNA2 together with crRNA 3. RNPs were obtained and transfected into cells as described above. The selected clones were analyzed by amplifying the genomic DNA with the following primers: F1: 5´-CAATAGTGGGGAAGCAGGAA-3´; F2: 5´-CCTTCCCGAATTTAGGCTTT-3´, and R1: 5´-TCCTGCTTCGAAGTCTCGTT-3´, used in two combinations, F1-R1 and F2-R1. The resulting PCR amplicons were sequenced to confirm the mutations introduced in the *TLR3* gene.

Following the same approach, we also generated DF-1 RIPK1 KO cell lines. Two sequence-specific crRNAs were designed to generate a large deletion encompassing from exon 2 to 12 in the *chRIPK1* gene, crRNA 1: 5´-GACAAACAACAATTAGATGCTGG-3´; crRNA 2: 5´- GATCACGACTACGAACGAGATGG-3´. Individual clones were selected and grown, and genetically characterized by amplifying the regions of genomic DNA around the site of interest with the following primers: F1: 5´-TATATGGCAGCGTCCCTCTC-3´ and R2**: 5´-**TTGGCAGAAAAATCCATTCC-3´. The resulting PCR amplicons were sequenced to confirm the mutations introduced in the *RIPK1* gene.

### Small interfering RNA transfection

Silencing of endogenous *MDA5* and *MAVS* genes in DF-1 cells was performed using specific siRNAs, along with the respective non-targeting control small interfering RNA (siRNA) (50 nM, Dharmacon™). Cells in 24-well plates were transfected with these siRNAs using Lipofectamine RNAiMax (Invitrogen), following the manufacturer´s instructions. The efficiency of silencing was checked by comparing mRNA expression levels of *MDA5* or *MAVS*, between silenced cells and control cells at different times pt by RT-qPCR. The corresponding siRNAs sequences were 5´-AAGAAGGGAUCCAUUUAGA-3´ (siMDA5), 5´-UGCUGCAGGAAGCUUUGAA-3´ (siMAVS) (Liniger et al., 2012), and 5´-AAGGACGCUGAGGCCUAAUCCUGUU-3´ (siControl) (Rajsbaum et al., 2014).

### Reverse Transcription-real time quantitative PCR

Total RNA was isolated by using the NucleoSpin RNA plus (Macherey-Nagel) according to the manufacturer´s instructions. Purified RNA (250 ng) was reverse transcribed into cDNA by using random primers (ThermoFisher Scientific) and SuperScript III reverse transcriptase (Invitrogen), according to the manufacturer´s protocol. The cDNA was then subjected to RT-qPCR using the gene-specific primers indicated in **Table 1**. RT-qPCRs were performed in duplicate using Power SYBR green PCR master mix (ThermoFisher Scientific), according to the manufacturer´s protocol, on an Applied Biosystems 7500 real-time PCR system instrument. Reactions were performed as follows: 2 min at 50°C; 10 min at 95°C; 40 cycles of 15 s at 95°C and 1 min at 60°C; and finally, 15 s at 95°C, 1 min at 60°C, 30 s at 95°C, and 15 s at 60°C to build the melt curve. Gene expression levels were normalized to the *Glyceraldehyde-3-Phosphate Dehydrogenase (GAPDH)* gene, and the results were calculated as fold changes in gene expression relative to mock-infected cells by using the delta-delta *C_T_*(threshold cycle) method of analysis. Dilutions of plasmids containing the sequence amplified by each set of primers run in parallel were used to establish the corresponding standard curves.

**Table 1:** List of primers used for RT-qPCR.

Gene	Forward primer (5´-3´)	Reverse primer (5´-3´)
*GAPDH*	ATCAAGAGGGTAGTGAAGGCTGCT	TCAAAGGTGGAGGAATGGCTGTCA
*IFNB*	ACCAGGATGCCAACTTCTCTTGGA	ATGGCTGCTTGCTTCTTGTCCTTG
*Mx*	TTCACGTCAATGTCCCAGCTTTGC	ATTGCTCAGGCGTTTACTTGCTCC
*OAS*	GCAGAAGAACTTTGTGAAGTGGC	TCGGCTTCAACATCTCCTTGTACC
*MDA5*	TGAAGGCAAAGAGAGATCAGCGTAAGA	CATATCAATTGTGGCAATTCTTGCACAGGA
*TLR3*	GGATCCATGGTGCAGGAAGTT	TCGACTTTGCTCAATAGCTTGCT
IBDV segment A	AAGGGCAGCTACGTCGATCTAC	TGGCAACTTCGTCTATGAAAGC

### Statistics

GraphPad Prism version 5.03 software (GraphPad Software, La Jolla, CA) was used to determine statistical significance, using the Student unpaired two-tailed *t* test.

## Results

### Interplay between IFN production and apoptosis induction during IBDV infection

**Figure 1:**
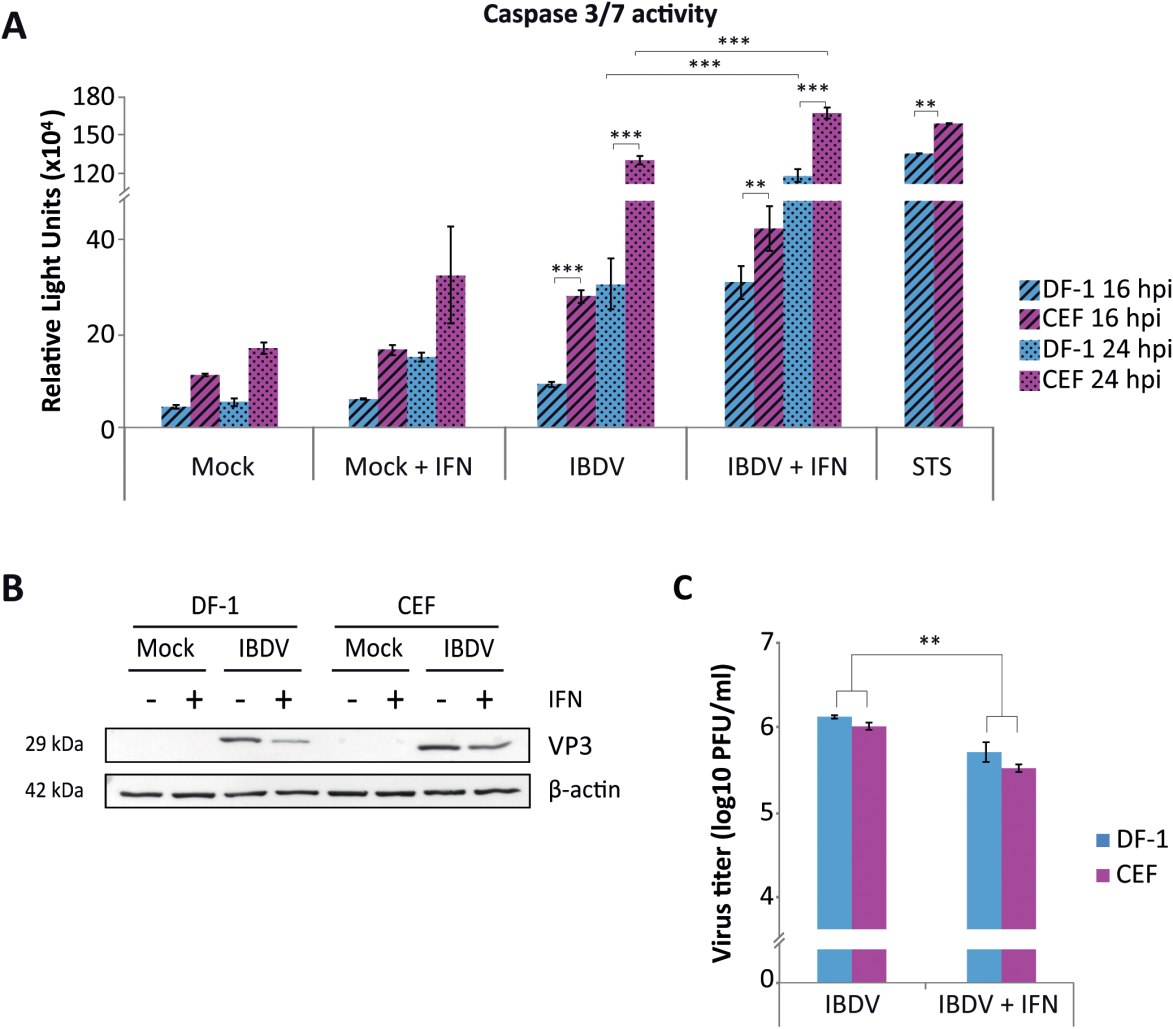
Induction of apoptosis by chIFN-α treatment in chicken DF-1 and CEF cells infected with IBDV. DF-1 and CEF cells mock-infected or infected with IBDV (MOI of 2 PFU/cell) were treated, or not, with chIFN-α (1,000 IU/ml) at 3 h pi, and collected at 16 and 24 h pi. **(A)** Apoptosis was measured by using the Caspase-Glo 3/7 assay kit in samples collected at 16 (striped bars) and 24 (dotted bars) h pi. A set of DF-1 and CEF cells were treated with 1 µM Staurosporine (STS) to compare the ability of these cells to undergo apoptosis in response to other stress inducing stimuli. Each determination was carried out in duplicate. **(B)** Immunoblot analysis of total cell extracts collected at 24 h pi with antibodies specifically recognizing the IBDV structural VP3 protein. Antibodies to β-actin were used for protein loading control. **(C)** Analysis of extracellular virus titers in samples collected at 24 h pi. Bars indicate means ± standard deviations based on data of duplicate samples from three independent experiments. ** and *** indicate p values of <0.01 and <0.001 respectively, as determined by unpaired Student´s test.

Previous research from our laboratory demonstrated that type I IFN plays a pivotal role in the fate of IBDV-infected cells. Specifically, while pretreatment of cells with IFN-α provides a robust protection against IBDV replication, its addition early after infection triggers a significant apoptotic response that effectively eliminates infected cell cultures. Although this effect was characterized in depth in human Hela cells, it was also confirmed to occur in avian DF-1 cells (Cubas-Gaona et al., 2018). This cell line comes from a spontaneously immortalized primary culture of chicken embryonic fibroblasts (CEF). Since its establishment, this cell line has been widely used as a model for studying infections caused by different avian viruses, including IBDV. However, DF-1 cells express higher levels of Suppressor of Cytokine Signalling 1 (SOCS1), which is known to attenuate the innate immune response (Kong et al., 2011; Giotis et al., 2017), than the progenitor CEF cells. Therefore, we considered it important to conduct a side-by-side comparative study of the effect of IFN during IBDV infection in CEF and DF-1 cells. For this, mock-infected or IBDV-infected DF-1 and CEF cells were treated with 1,000 U/mL of recombinant chIFN-α at 3 h pi. All infections throughout this study were performed at a MOI of 2 PFU/cell, and cells were collected at two time points, 16 and 24 h pi for subsequent analyses. In both DF-1 and CEF cells exogenous IFN treatment enhanced the cytopathic effect (CPE) caused by IBDV infection at either time pi when compared with untreated infected cells, and these morphological changes corresponded to apoptotic cell death as determined by analyzing the activity of caspases 3 and 7 in the cultures with the commercial Caspase-Glo 3/7 kit (**Figure 1A**). Although similar apoptosis trend along the infection was observed in both cell types, either in IFN-treated and in untreated cells, higher levels of caspase 3/7 were observed in CEF cells at each time point pi. These differences could not be attributed to differences in the extent of the infection since the results of accumulation of the viral protein VP3 by Western blot (**Figure 1B**) and the titers of virus in the culture medium (**Figure 1C**) were similar for both cell types. Based on these results, we conclude that IBDV infection *per se* causes apoptosis in both chicken cell types at late times pi, but early treatment with chIFN-α in IBDV-infected cells significantly increases the apoptotic response in both cell types. Significantly, similar results were also observed in chicken B cells from the immortalized DT40 cell line (Data not shown).

**Figure 2:**
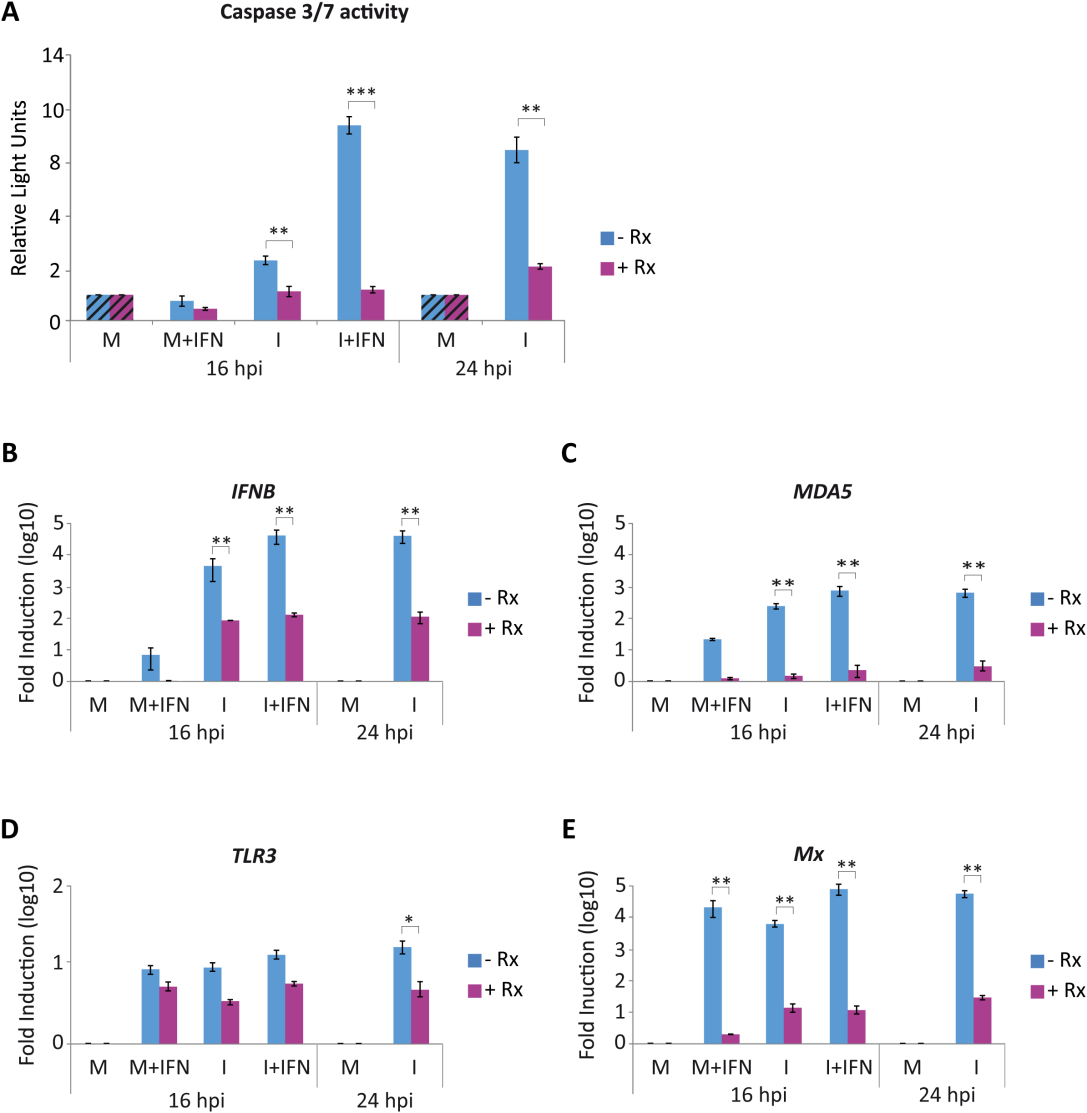
Treatment with the JAK/STAT inhibitor Ruxolitinib (Rx) significant decrease apoptotic death of IBDV-infected DF-1 cells. DF-1 cells mock-infected (M) or infected with IBDV (MOI of 2 PFU/cell) (I) were treated with Rx (4 µM) after virus adsorption, then treated or not with chIFN-α (1,000 IU/ml) at 3 h pi (M+IFN, I+IFN), and collected at 16 and 24 h pi. **(A)** Apoptosis was measured by using the Caspase-Glo 3/7 assay kit. Each determination was carried out in duplicate. Caspase values from infected cell samples were normalized to those from mock-infected cells (M) (striped bars). **(B-E)** The expression levels of *IFNB* **(B)**, *MDA5* **(C)**, *TLR3* **(D)** and *Mx* **(E)** genes were determined by SYBR green-based RT-qPCR. Recorded values for each cellular gene were normalized to the *GAPDH* mRNA content and presented on a log_10_ scale as the fold induction over the level found in mock-infected DF-1 cells. Bars indicate means ± standard deviations based on data of duplicate samples from three independent experiments. *, ** and *** indicate *p* values of <0.05, <0.01 and <0.001 respectively, as determined by unpaired Student´s test.

Recent research has demonstrated that the inactivation of the JAK/STAT pathway markedly attenuates IBDV-induced cell death in DF-1 cells, which in turn, facilitates the establishment of persistent IBDV infections (Broto et al., 2021). In this study, we employed Ruxolitinib (Rx), a selective inhibitor of the JAK/STAT signaling pathway, to assess the contribution of endogenous type I IFN to the apoptotic response observed in IBDV-infected DF-1 cells that result in the extensive cell death observed late in infection. To this end, mock-infected or IBDV-infected DF-1 cells were treated 1 h after virus adsorption with Rx at a concentration of 4 μM and cells were collected at 16 and 24 h pi. In parallel, additional sets of mock-infected and IBDV-infected cultures were treated with 1,000 U/mL of recombinant chIFN-α at 3 h pi and harvested at 16 h pi. As shown above, the results demonstrated that the levels of apoptosis observed in cells infected for 24 h were comparable to those observed in IFN-treated infected cells collected at 16 h pi. (**Figure 2A**). In both cases, apoptosis was significantly diminished in Rx-treated cell cultures. Then, the expression of the *IFNB* gene in these samples was analyzed by RT-qPCR to explore the relationship between IFN signaling and apoptosis. As shown in **Figure 2B**, high levels of induction of *IFNB* gene were observed in infected DF-1 cells harvested at 24 h pi, being comparable to those detected in IFN-treated infected cells harvested at 16 h pi. However, in all infected cells treated with Rx there was a notable reduction in the expression of this gene (ca. 3 log units). Similar expression patterns were observed for *MDA5*, *TLR3* and *Mx* (**Figures 2C–E**). These data, indicate the existence of a feedback mechanism whereby IFN-β, secreted by infected cells, stimulates the expression of the gene itself as well as those of the ISGs. The use of Rx confirms that inhibiting the type I IFN signaling pathway results in a significant reduction in apoptosis in IBDV-infected cells, suggesting that IFN released by IBDV-infected cells is responsible for the apoptosis observed at late times pi.

### Apoptosis of IBDV-infected cells is dependent upon viral replication

**Figure 3:**
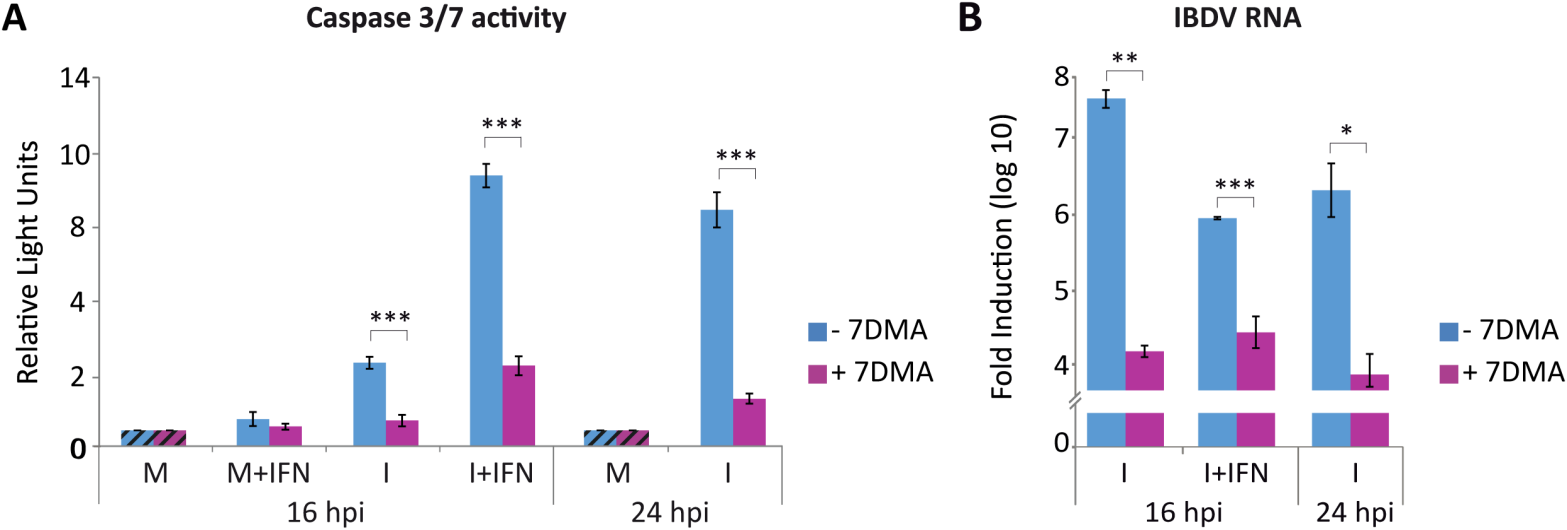
The viral polymerase inhibitor 7DMA significantly reduced apoptotic cell death in IBDV-infected DF-1 cells. DF-1 cells mock-infected (M) or infected with IBDV (MOI of 2 PFU/cell) (I) were treated with 7DMA (0.2 mM) after virus adsorption, then treated or not with chIFN-α (1,000 IU/ml) at 3 h pi (M+IFN, I+IFN), and collected at 16 and 24 h pi. **(A)** Apoptosis was measured by using the Caspase-Glo 3/7 assay kit, and as above, caspase values from infected cell samples were normalized to those from mock-infected cells (M) (striped bars). **(B)** The accumulation levels of the IBDV RNA were determined by SYBR green-based RT-qPCR. Bars indicate means ± standard deviations based on data of duplicate samples from three independent experiments. *, ** and *** indicate *p* values of <0.05, <0.01 and <0.001 respectively, as determined by unpaired Student´s test.

Previous results obtained in HeLa cells indicated that viral RNA replication/transcription is required for apoptosis induction upon IFN treatment of IBDV-infected human HeLa cells (Cubas-Gaona et al., 2018). Then, to confirm that viral RNA replication/transcription is also required in the context of IBDV-infected chicken cells we used the viral RNA polymerase inhibitor 7-deaza-2´-C-metiladenosina (7DMA). For this, mock-infected or cells infected with IBDV were treated 1 h after virus-adsorption with 7DMA at a concentration of 0.2 mM and collected at 16 and 24 h pi for analysis. As above, a portion of the cultures collected at 16 h pi had been treated with chIFN-α (1,000 U/ml) at 3 h pi. A drastic decrease in caspase 3/7 activity was detected in samples from 7DMA-treated cells, both in those from cultures just infected and harvested at 16 and 24 h pi, as well as in samples from IFN-treated cell cultures, compared to cells not treated with 7DMA (**Figure 3A**). The effectiveness of the 7DMA treatment was assessed by the analysis of viral RNA levels by RT-qPCR. As shown in **Figure 3B**, an overall reduction of ca. 2 to 3 log units was determined in all samples treated with 7DMA. These results indicate that the replication/transcription process of viral dsRNA is a critical determinant for apoptosis induction also in IBDV-infected DF-1 cells.

### MDA5 and MAVS contribute to the activation of apoptosis in IBDV-infected DF-1 cells

**Figure 4:**
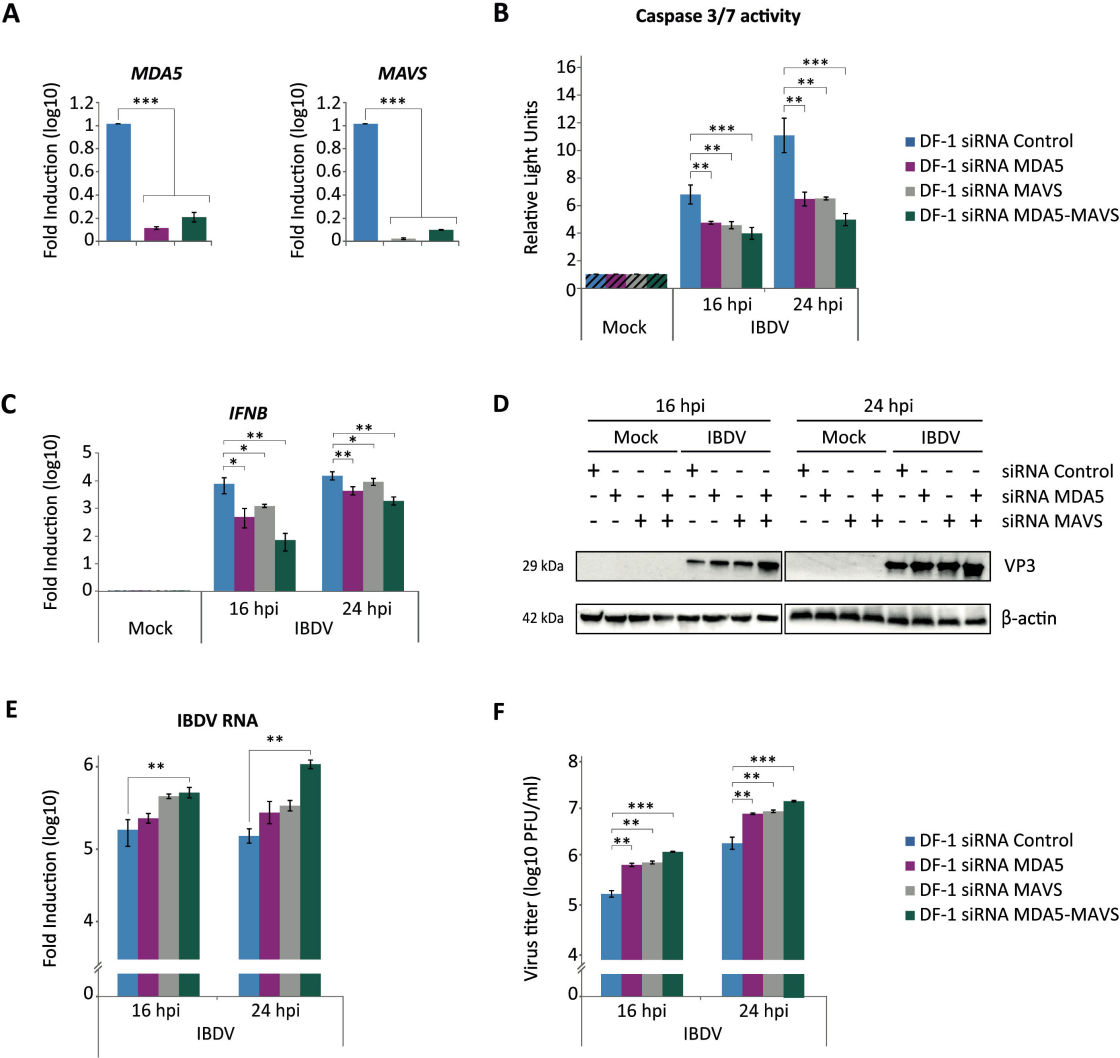
MDA5 and MAVS contribute to induce apoptosis in DF-1 cells infected with IBDV. DF-1 cells were transfected with siRNAs that silence the expression of MDA5 and MAVS proteins or with a control siRNA. At 24 h pt, cell cultures were infected or not (Mock) with IBDV (MOI 2 PFU/cell), and cell samples were collected at 16 and 24 h pi. **(A)** Extracts from uninfected cells collected at 24 h pt were used for total RNA extraction and subsequent analysis of *MDA5* and *MAVS* mRNA levels by RT-qPCR using specific primers for the silenced genes. The expression values of the *MDA5* and *MAVS* genes were normalized to the *GAPDH* gene. Values were relative to those obtained in cultures transfected with the control siRNA. **(B)** Apoptosis was measured by using the Caspase-Glo 3/7 assay kit, and each determination was carried out in duplicate. Caspase values from infected cell samples were normalized to those from mock-infected cells (Mock) (striped bars). **(C)** RT-qPCR study of the expression of *IFNB* gene. Cellular gene expression values were normalized to those of the *GAPDH* mRNA content and are presented on a log_10_ scale as the fold induction over the level found in mock-infected DF-1 silenced control cells. **(D)** Immunoblot analysis of total cell extracts with antibodies specifically recognizing the IBDV structural VP3 protein. Antibodies to β-actin were used for protein loading control. **(E)** The accumulation levels of the IBDV RNA were determined by SYBR green-based RT-qPCR. **(F)** Extracellular virus yields. Bars indicate means ± standard deviations based on data of duplicate samples from three independent experiments. *, ** and *** indicate *p* values of <0.05, <0.01 and <0.001, respectively, as determined by unpaired Student´s test.

As mentioned above, results from different laboratories, including ours, showed that in chicken cells IBDV dsRNA is recognized by the cytoplasmic sensor MDA5 leading to the recruitment of the adaptor protein MAVS and the activation of the IFN signaling pathway. We therefore set out to investigate the possible involvement of MDA5 and MAVS proteins in the apoptotic process triggered during IBDV infection by using the RNA interference-mediated gene silencing technology. After optimizing transfection conditions (data not shown), DF-1 cells were transfected with specific siRNAs targeting *MDA5* and *MAVS* genes, either independently or simultaneously, as described in the Materials and Methods section. Under optimal conditions, we achieved a 75-90% reduction in the mRNA expression of both genes compared to cells transfected with a control siRNA (**Figure 4A**).

At 24 h pt, silenced DF-1 cells were infected with IBDV and harvested at 16 and 24 h pi. Phase-contrast microscopy revealed a marked reduction in the CPE in *MDA5*- or *MAVS*-silenced cells compared to control cells at both time points (data not shown). This observation was corroborated by the analysis of caspases 3/7 activation, which revealed a reduction in apoptosis of approximately 1.5-fold in cells silenced with either *MDA5* or *MAVS* siRNAs at 24 h pi. Notably, co-silencing of both genes resulted in a reduction of ca. 2-fold compared to cells silenced with the control siRNA (**Figure 4B**). Consistently, RT-qPCR analysis of *IFNB* gene expression showed significantly reduced transcript levels in all siRNA-treated infected cultures, with the strongest suppression observed in the double-silenced condition (**Figure 4C**).

Regarding IBDV replication, a notable increase in the accumulation of the viral VP3 protein was observed in DF-1 cells transfected with *MDA5* or *MAVS* siRNAs, with a further enhancement in cells transfected with both siRNAs (**Figure 4D**). These results were then complemented with the quantification of viral RNA accumulation by RT-qPCR and the determination of viral titers in cell supernatants. Both, the accumulation of IBDV RNA in cells and the titers of the virus in the culture supernatants, were found to be significantly higher in samples from silenced cells with either *MDA5* or *MAVS* siRNA, at both 16 and 24 h pi, compared to cells infected after transfection with control siRNA. Again, this difference was accentuated when both siRNAs were used simultaneously (**Figures 4E, F**).

Collectively, these results indicate that silencing *MDA5* and *MAVS* genes in DF-1 cells prior to IBDV infection results in a significant, but partial reduction in the activation of apoptosis, a decrease in the activation of the innate immune response and an increase in the efficiency of viral replication.

### PKR contributes significantly to apoptosis induction in IBDV-infected DF-1 cells

**Figure 5:**
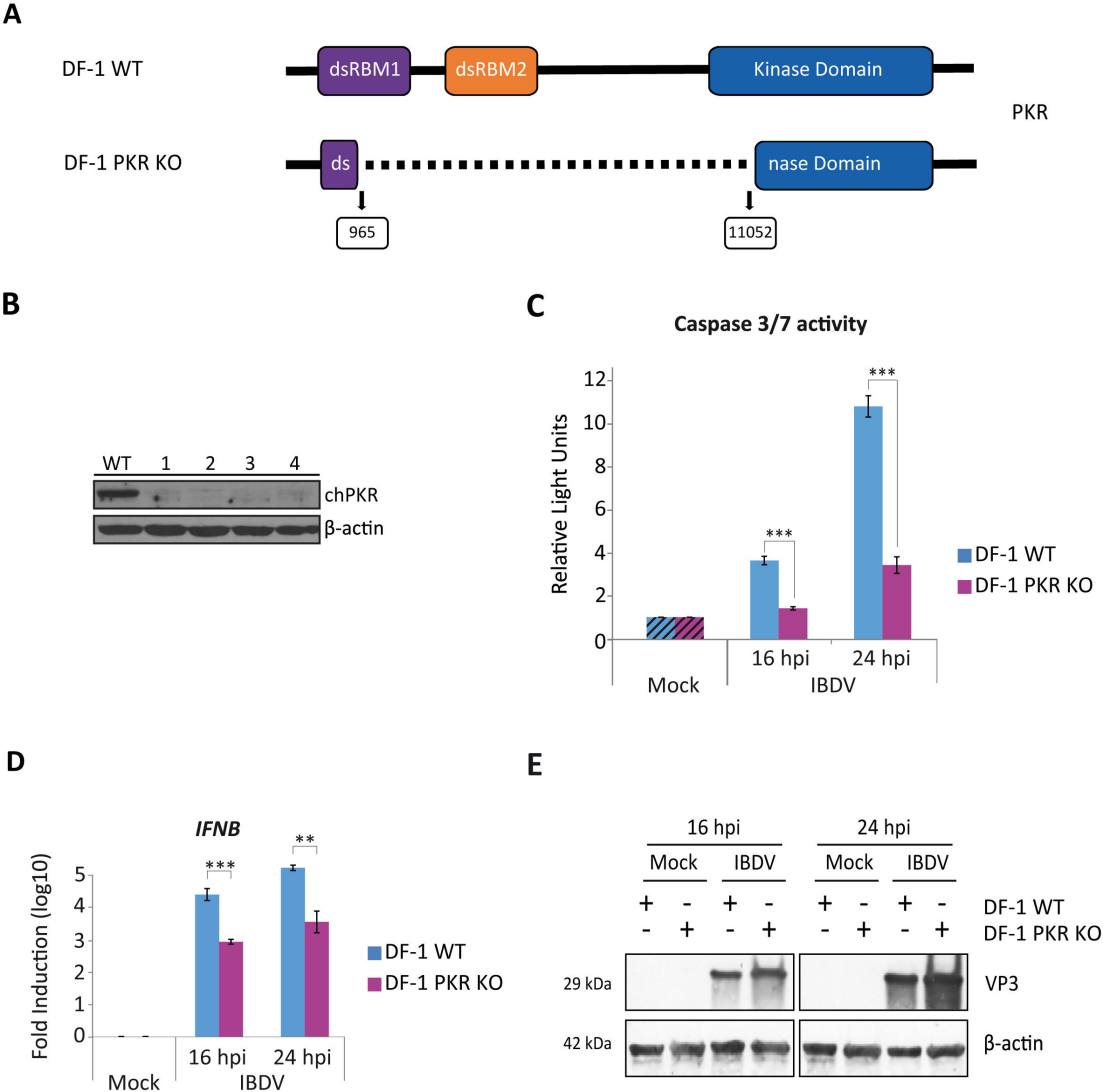
The induction of apoptosis by IFN in IBDV-infected DF-1 cells is partially dependent on PKR expression. **(A)** Schematic representation of the structure of the WT *PKR* gene and the deleted version of the gene present in the DF-1 PKR KO cells. The boxed numbers below the scheme indicate the nucleotide positions at which the deletions occurred. **(B)** Western blot analysis of total cell extracts with antibodies specifically recognizing the PKR protein. Antibodies to β-actin were used for protein loading control. **(C-E)** DF-1 and DF-1 PKR KO cells were mock-infected or infected with IBDV (MOI of 2 PFU/cell) and at 16 and 24 h pi the levels of apoptosis **(C)** and the expression of *IFNB* gene **(D)** were analyzed by using the Caspase-Glo 3/7 assay kit and RT-qPCR, respectively. Caspase activity was determined in duplicate, and the values normalized to those from mock-infected cells (Mock) (Striped bars). Cellular gene expression values were normalized to those of the *GAPDH* mRNA content and are presented on a log_10_ scale as the fold induction over the level found in mock-infected DF-1 or DF-1 PKR KO cells. **(E)** Immunoblot analysis of total cell extracts with antibodies specifically recognizing the IBDV structural VP3 protein. Antibodies to β-actin were used for protein loading control. Bars indicate means ± standard deviations based on data of duplicate samples from three independent experiments. ** and *** indicate *p* values of <0.01 and <0.001, respectively, as determined by unpaired Student´s test.

PKR plays a pivotal role in cellular responses to diverse stress-inducing stimuli, including viral infections, and is involved in apoptosis induction through various mechanisms (Kaufman, 1999; McAllister and Samuel, 2009). In this regard, we previously showed that PKR acts as a critical mediator of the apoptotic response in IBDV-infected human HeLa cells treated with IFN-α (Cubas-Gaona et al., 2018). Then, we sought to study the potential contribution of PKR to apoptosis triggering in IBDV-infected DF-1 cells. For this, we generated a DF-1 PKR KO cell line using the CRISPR/Cas9 technology as described in the Materials and Methods section. We designed two guides to produce a double cut in the target gene that should result in a large deletion entailing the loss of the sequence coding for the two N-terminal dsRNA binding domains and a portion of the active site of the protein, as depicted in **Figure 5A**. Four cell clones were selected, and the expression of PKR was assessed by Western blot analysis in comparison with DF-1 WT cells. No expression of PKR was detected in any of the clones, while the protein present in WT cells was clearly detected (**Figure 5B**). To further characterize these cells, genomic DNA was extracted and subjected to sequencing analysis, which revealed that only clone 3 presents a deletion of the expected size (10,107 Kb), while in the other three clones there had occurred rearrangements of the sequence most likely resulting from non-homologous recombination repair. DF-1 PKR KO cells from clone 3 were then used to determine the role of PKR in apoptosis induction in IBDV-infected chicken cells. For this, WT and DF-1 PKR KO cells were infected and samples were taken at 16 and 24 h pi for the analysis of apoptotic cell death by determining caspase 3/7 activity. We observed a significant decrease in the apoptotic levels in DF-1 PKR KO as compared with DF-1 WT cells, ranging from about 30% reduction at 16 h pi to 60% at 24 h pi (**Figure 5C**). Furthermore, RT-qPCR analysis showed a concomitant reduction in *IFNB* gene expression in IBDV-infected DF-1 PKR KO cells compared with DF-1 WT cells at both time points (**Figure 5D**). Consistent with this decreased IFN response, DF-1 PKR KO cells also exhibited a notable increase in VP3 protein accumulation at both time points pi as determined by Western blot (**Figure 5E**), indicating enhanced IBDV replication. These results show that PKR plays a major role in the induction of apoptosis upon IBDV infection, that could be directly or indirectly related with IFN-β production. However, the persistence of a significant level of apoptotic cell death in the absence of the PKR protein indicates that other factor(s) is also involved in IBDV-induced apoptosis in chicken cells.

### TLR3 is an essential factor in the induction of apoptosis in IBDV-infected DF-1 cells

**Figure 6:**
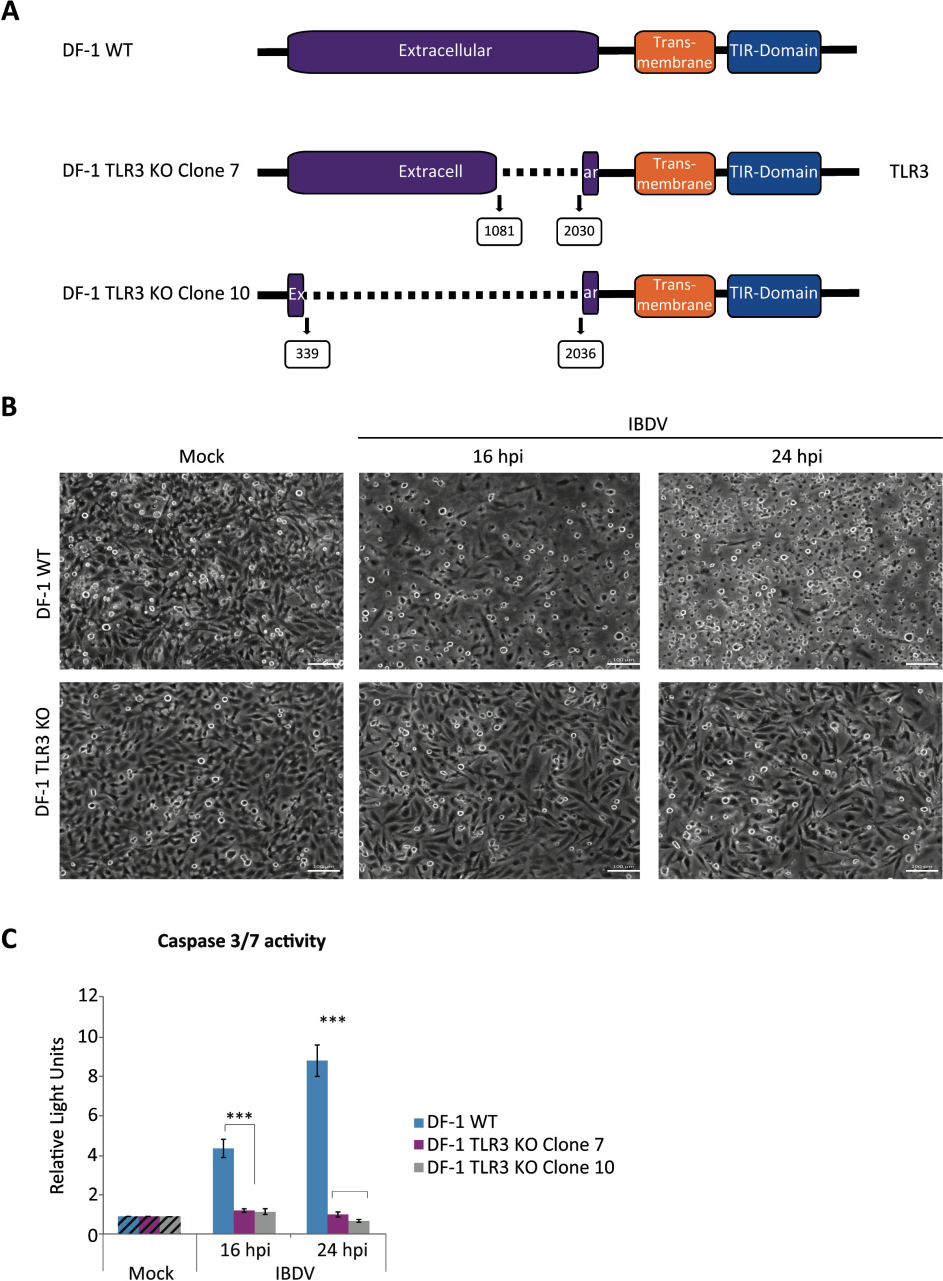
Apoptosis triggering in IBDV-infected DF-1 cells is dependent on TLR3 expression. **(A)** Schematic representation of the structure of the *TLR3* gene and the deleted versions of the gene present in two selected DF-1 TLR3 KO cell clones. The boxed numbers below the schemes indicate the nucleotide positions at which the deletions occurred. **(B, C)** DF-1 and DF-1 TLR3 KO cells were mock-infected or infected with IBDV (MOI of 2 PFU/cell) and at 16 and 24 h pi the extent of CPE **(B)** and the levels of apoptosis **(C)** were analyzed by phase-contrast microscopy and using the Caspase-Glo 3/7 assay kit, respectively. The images of DF-1 TLR3 KO cells shown in panel B are from clone 10. Caspase activity was determined in duplicate samples, and the values normalized to those from mock-infected cells (Mock) (Striped bars). Bars indicate means ± standard deviations based on data of duplicate samples from three independent experiments. *** indicate *p* value of <0.001, as determined by unpaired Student´s test.

As introduced earlier, TLR3 is a transmembrane receptor located mainly on endosomes, where it recognizes viral dsRNA and initiates innate immune signaling cascades. It has been proposed that IBDV enters the cell through a macropinocytosis mechanism (Gimenez et al., 2015) and internalizes into endosomal vesicles where the virus capsid is destabilized and RNPs are released. It is therefore reasonable to assume that the TLR3 receptor might act as the first cellular sensor capable of recognizing IBDV dsRNA upon virus entry into the cells. This, together with the observation that *TLR3* gene expression is induced in IBDV-infected DF-1 cells, as well as in bursal cells from infected animals (Rauf et al., 2011; Guo et al., 2012; Smith et al., 2015; Lee et al., 2015a; He et al., 2017), led us to investigate the role of this transmembrane receptor during the IBDV infection process. For this, we generated TLR3-deficient DF-1 cell lines using the CRISPR/Cas9 technology, employing two paired combinations of three guide RNAs, that allowed us to generate two different partial deletions affecting the extracellular domain of the protein responsible for dsRNA recognition. As depicted in **Figure 6A**, two representative cell clones, 7 and 10, were selected. Sequencing analysis revealed that clone 7 harbors a 949 bp (nucleotides 1081-2030) deletion in the *TLR3* gene, causing frameshifts and the predicted expression of a defective protein, while clone 10 presents a deletion of 1,697 bp (nucleotides 339-2036) affecting most of the extracellular domain.

Once the DF-1 TLR3 KO cell lines were generated, our objective was to ascertain the effect of the TLR3 protein ablation on the IBDV infection process. To this end, DF-1 WT and DF-1 TLR3 KO cells from both clones were infected with IBDV and samples were taken at 16 and 24 h pi for various analyses. Phase contrast microscopy revealed a significant reduction in the CPE of the infected DF-1 TLR3 KO cells, particularly at 24 h pi (**Figure 6B**). This observation was corroborated by the analysis of caspase 3/7 activity. As shown in **Figure 6C**, there was a drastic reduction in the level of apoptosis detected in infected cells from both TLR3 KO cell lines in comparison to DF-1 WT cells at both times pi. However, the impact was more pronounced at 24 h pi, when higher levels of apoptosis are detected in DF-1 WT cells. Indeed, the levels of apoptosis in the two DF-1 TLR3 KO cell lines infected with IBDV are comparable to those found in uninfected (mock) cells, as well as to those detected in WT infected cells treated with the pan-caspase inhibitor Z-VAD-FMK (**S1 Figure**). However, both cell lines undergo high levels of apoptosis, comparable to those of DF-1 WT cells, after treatment with Staurosporine, a well-known apoptotic inducer used as a control (**S1 Figure**). Strikingly, these findings demonstrate that TLR3 silencing alone is sufficient to fully abrogate apoptosis induced by IBDV infection in DF-1 cells, underscoring its pivotal role in orchestrating the apoptotic response.

### Role of TLR3 in the control of viral replication and dissemination

**Figure 7:**
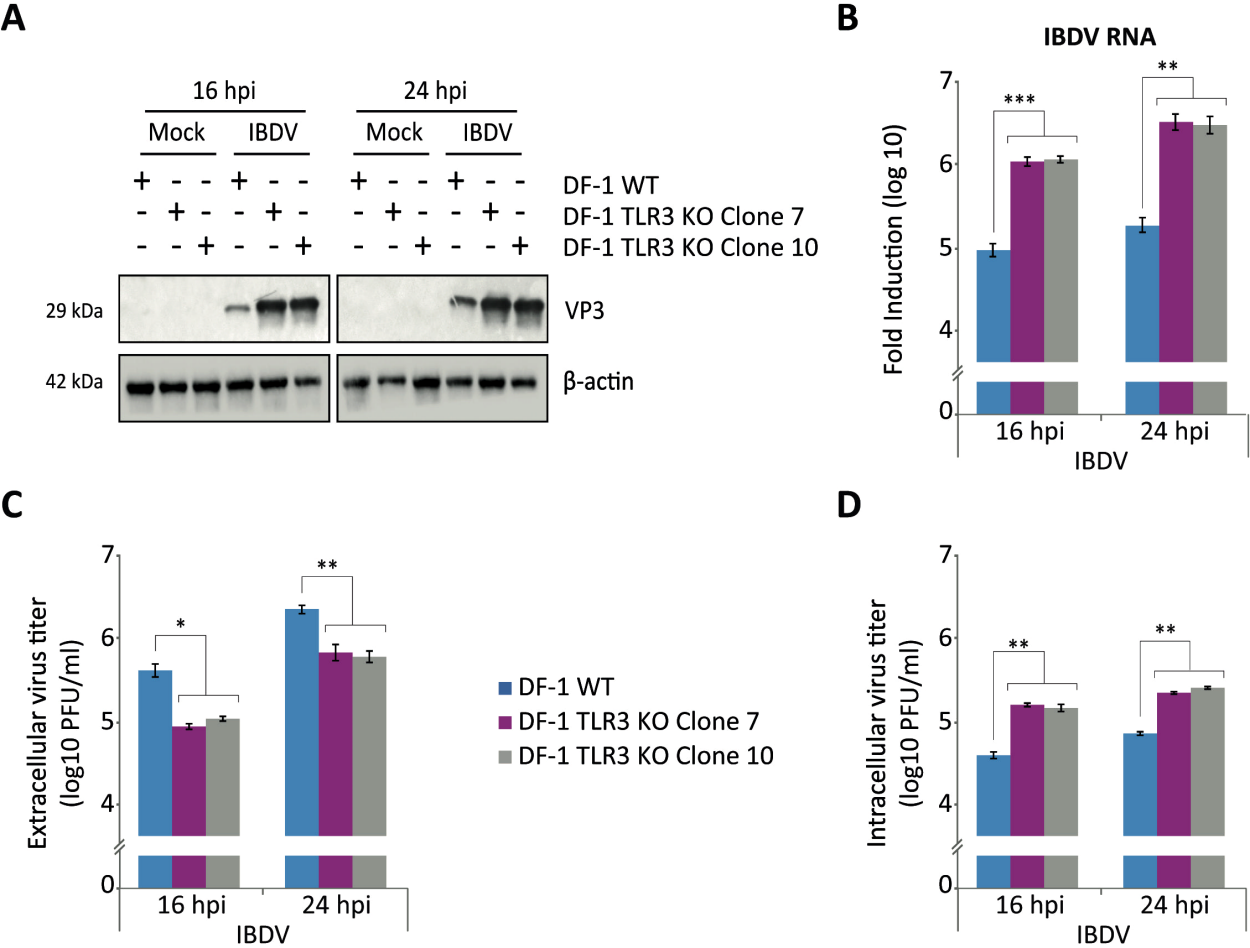
TLR3 is an essential factor in the control of IBDV replication. DF-1 and DF-1 TLR3 KO cells were mock-infected or infected with IBDV (MOI of 2 PFU/cell). Cell samples were harvested at 16 and 24 h pi. **(A)** Western blot analysis of total cell extracts with antibodies specifically recognizing the IBDV structural VP3 protein. Antibodies to β-actin were used for protein loading control. **(B)** The accumulation of the IBDV RNA was determined by SYBR green-based RT-qPCR. **(C, D)** Analysis of extracellular **(C)** and intracellular **(D)** virus titers. Bars indicate means ± standard deviations based on data of duplicate samples from three independent experiments. *, ** and *** indicate *p* values of <0.05, <0.01 and <0.001, respectively, as determined by unpaired Student´s test.

We next wanted to analyze the impact of the TLR3 deficiency on IBDV replication. Using the same infection conditions described above, we analyzed the accumulation of the viral protein VP3 by Western blot. A significant increase in VP3 accumulation was clearly observed in samples obtained from both DF-1 TLR3 KO cell lines compared to DF-1 WT cells at both time points pi (**Figure 7A**). To confirm these findings, we performed an RT-qPCR analysis to assess the accumulation of viral RNA. We found that in the absence of TLR3, the amount of viral RNA increased by more than 1-log unit at both times pi (**Figure 7B**). However, the analysis of the accumulation of infectious virus in the culture supernatants revealed a significant decrease in viral titers obtained in samples from the DF-1 TLR3 KO cell lines compared to the parental cells (**Figure 7C**). These results appear to indicate a reduction in viral replication and are therefore in apparent contradiction with the findings from the VP3 protein analysis by Western blot and the quantification of viral RNA by RT-qPCR. To try to solve this discrepancy, we quantified the intracellular virus titers in a different repeated set of infected cultures. The results showed that the intracellular viral titers obtained from the DF-1 TLR3 KO cells were significantly higher than those obtained from the DF-1 WT cells at both time points pi (**Figure 7D**), concurring with the protein and RNA data. Taken together, this set of data demonstrate that TLR3 is a key player in the apoptotic process triggered during IBDV infection in DF-1 cells, as well as in IBDV replication. In addition, a potential role in virus release is suggested by the reduction of extracellular viral titers observed in the DF-1 TLR3 KO cells.

As the results obtained with both DF-1 TLR3 KO cell lines are similar, we decided to use only clone 10 for further analyses.

### The TLR3-initiated pathway is primarily responsible for IFN production in IBDV-infected DF-1 cells

**Figure 8:**
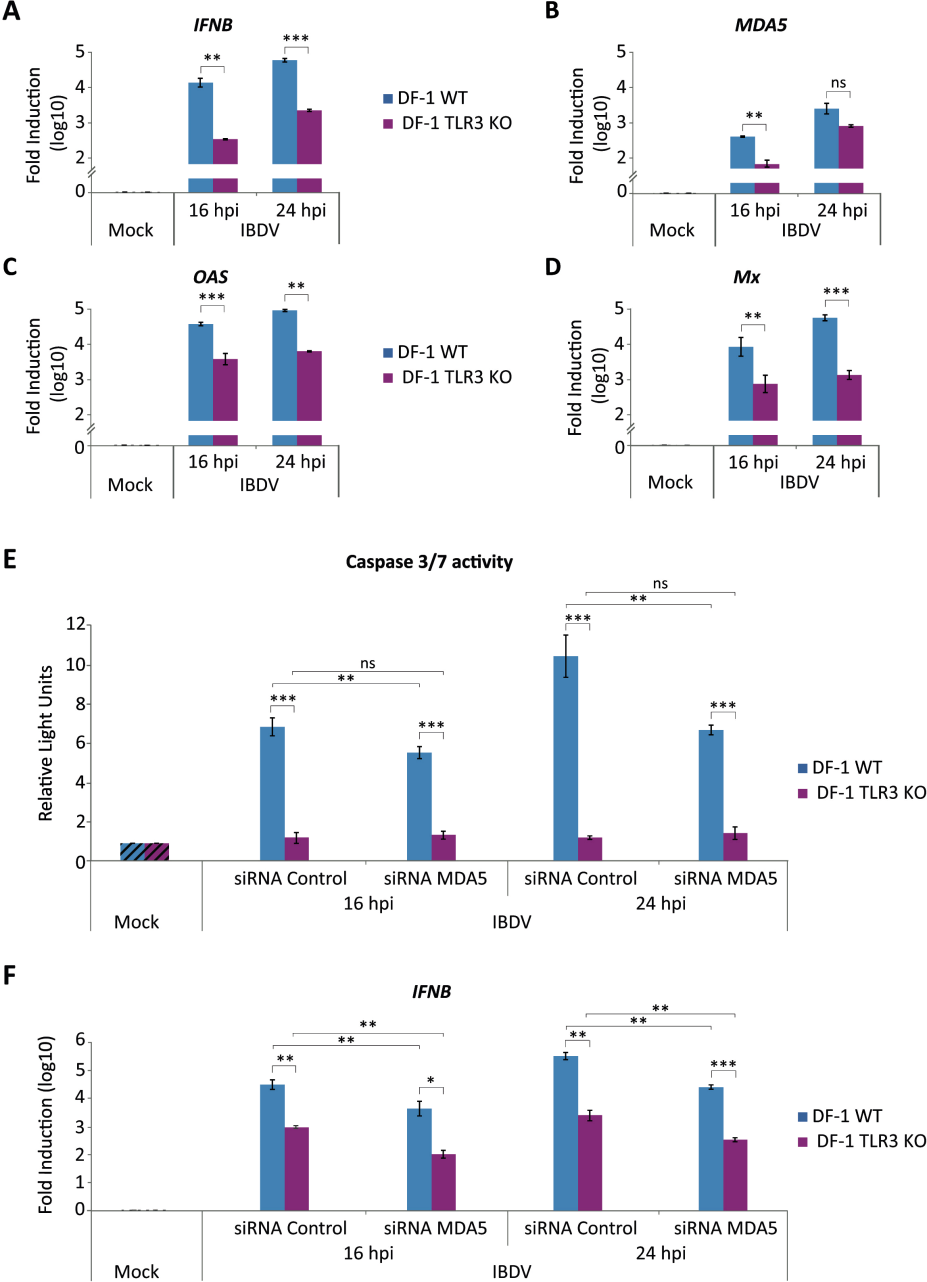
Activation of the innate immune response during IBDV infection in DF-1 cells. **(A-D)** DF-1 and DF-1 TLR3 KO cells were mock-infected or infected with IBDV (MOI of 2 PFU/cells) and cells were harvested at 16 and 24 h pi for RNA extraction and RT-qPCR analysis. The expression levels of *IFNB* **(A)**, *MDA5* **(B)**, *OAS* **(C)** and *Mx* **(D)** genes were determined by SYBR green-based RT-qPCR. Recorded values for each cellular gene were normalized to the *GAPDH* mRNA content and are presented on a log_10_ scale as the fold induction over the level found in mock-infected DF-1 or DF-1 TLR3 KO cells. **(E, F)** DF-1 cells were transfected with a siRNA specifically silencing the expression of MDA5 protein, or with a control siRNA. At 24 h pt, cells were either infected or not (Mock) with IBDV (MOI 2 PFU/cell). Samples were collected at 16 and 24 h pi and the levels of apoptosis **(E)** and the expression of the *IFNB* gene **(F)** analyzed by using the Caspase-Glo 3/7 assay kit and RT-qPCR, respectively. Caspase activity was determined in duplicate, and the values normalized to those from mock-infected cells (Mock) (Striped bars). Cellular gene expression values were normalized to those of the *GAPDH* mRNA content and are presented on a log_10_ scale as the fold induction over the level found in mock-infected DF-1 or DF-1 TLR3 KO cells. Bars indicate means ± standard deviations based on data of duplicate samples from three independent experiments. *, ** and *** indicate *p* values of <0.05, <0.01 and <0.001, respectively, as determined by unpaired Student´s test. ns, not significant.

The next objective was to analyze *IFNB* gene expression during IBDV infection in DF-1 TLR3 KO cells. DF-1 WT and DF-1 TLR3 KO cells were infected and total RNA was isolated at 16 and 24 h pi for RT-qPCR analysis. As shown in **Figure 8A**, the induction of *IFNB* was significantly lower (ca. 2 log units) in samples from DF-1 TLR3 KO cells than in DF-1 WT cells at both time points pi. To further assess the impact of TLR3 deficiency on the antiviral response, we expanded the study to analyze the expression of several ISGs, including *MDA5*, *OAS* and *Mx*. The results confirmed the reduced innate immune response during the IBDV infection process in DF-1 TLR3 KO cells at both 16 and 24 h pi compared to parental cells (**Figures 8B–D**).

Then, we wanted to investigate the combined effect of TLR3 and MDA5 on the activation of the innate immune response induced in IBDV-infected DF-1 cells. To achieve this, we transfected DF-1 WT and DF-1 TLR3 KO cells with a MDA5-specific siRNA. Once we had confirmed that the silencing process had worked correctly, with a reduction of approximately 70% in *MDA5* mRNA compared to cells transfected with the control siRNA (data not shown), we proceeded to infect the cells with IBDV. Samples were taken at 16 and 24 h pi to quantify apoptosis and *IFNB* gene induction. The analysis of caspase 3/7 activation showed a significant reduction of apoptosis in DF-1 WT cells transfected with *MDA5* siRNA in comparison to samples that had been transfected with control siRNA, as we had previously observed (**Figure 3B**). However, no significant differences were identified in samples from DF-1 TLR3 KO cells transfected with *MDA5* siRNA or control siRNA at either 16 or 24 h pi (**Figure 8E**), as it could be expected since caspase 3/7 activity was already almost undetectable in non-transfected DF-1 TLR3 KO cells. Regarding *IFNB* gene transcription quantified by RT-qPCR, the results showed again that TLR3 deletion resulted in a significant decrease in *IFNB* mRNA levels in comparison to DF-1 WT cells (approximately 1.5 and 2 log units at 16 h pi and 24 h pi, respectively) (**Figure 8F**). Furthermore, the data showed a lower, but significant, decline in *IFNB* induction in both DF-1 WT and DF-1 TLR3 KO cells transfected with *MDA5* siRNA, compared to the same cells transfected with control siRNA at 16 and 24 h pi (≤1 log unit) (**Figure 8F**). Taken together, these results indicate a synergistic effect between MDA5 and TLR3-mediated innate immune response activation pathways in response to IBDV infection. While TLR3 appears to be the dominant contributor, MDA5 also plays a complementary role in modulating both antiviral gene expression and apoptosis.

### Involvement of TLR3 in IFN-β and NF-κB promoter activation by dsRNA

**Figure 9:**
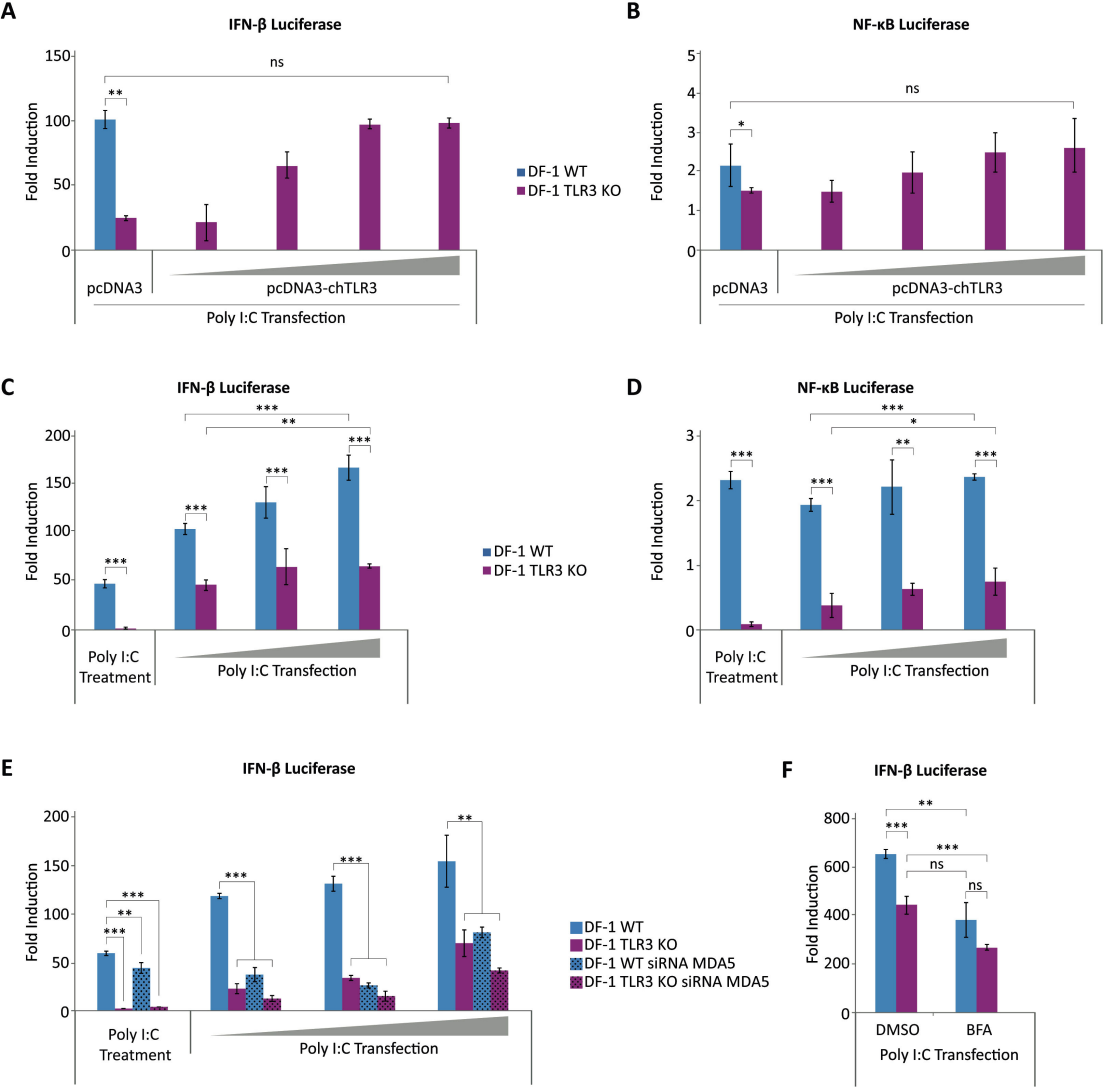
TLR3 is involved in the activation of IFN-β and NF-κB promoters by dsRNA in DF-1 cells. **(A, B)** IFN-β and NF-κB promoter activities are downregulated in DF-1 TLR3 KO cells but can be rescued by exogenous expression of TLR3. DF-1 and DF-1 TLR3 KO cells were co-transfected with pLucter (100 ng) and pR-null (30 ng) plasmids **(A)**, or with pSI-chNFκB-Luc (50 ng) plasmid **(B)** together with different amounts (100, 200, 400 or 800 ng) of the plasmid expressing chTLR3-His. At 8 h pt the cells were transfected with 250 ng of Poly I:C, and harvested at 24 h after plasmid transfection. A control consisting in cells co-transfected with pLucter, pR-null and the empty pCDNA3, or with the pSI-chNFκB-Luc, and the empty pCDNA3 plasmid (800 ng) was included in each assay. The samples were analyzed by the dual luciferase assay kit. Each determination was carried out in duplicate, and the firefly luciferase expression level of each sample was normalized by *Renilla* values. Results are expressed as the fold induction over the level found in the control DF-1 or DF-1 TLR3 KO cells not transfected with Poly I:C. **(C, D)** DF-1 and DF-1 TLR3 KO cells were transfected with the plasmids pLucter (100 ng) and pR-null (30 ng) **(C)** or with the plasmid pSI-chNFκB-Luc (50 ng) **(D)**. At 8 h pt, cells were either treated with Poly I:C (20 µg) added directly to the culture medium or transfected with increasing amounts (100, 200 and 300 ng) of Poly I:C. The samples were harvested at 24 h after plasmid transfection and used for luciferase activity quantification. **(E)** DF-1 and DF-1 TLR3 KO cells were transfected with a siRNA specifically silencing the expression of MDA5 protein, or with a control siRNA. At 24 h pt, cells were transfected with the plasmids pLucter (100 ng) and pR-null (30 ng) and 8 h later were either treated with Poly I:C (20μg) or transfected with increasing amounts (100, 200 and 300 ng) of Poly I:C. The samples were harvested at 24 h after plasmid transfection and used for luciferase assay. **(F)** DF-1 and DF-1 TLR3 KO cells were transfected with the plasmids pLucter (100 ng) and pR-null (30 ng). At 8 h pt, cells were transfected with 250 ng of Poly I:C and subsequently treated with BFA (50μM) or the vehicle DMSO 1 h later. The samples were harvested at 24 h after plasmid transfection and analyzed using the dual luciferase assay kit. In panels C-F values are presented as the fold induction over the level found in the control DF-1 or DF-1 TLR3 KO cells not treated with Poly I:C. Bars indicate means ± standard deviations based on data of duplicate samples from three independent experiments. *, ** and *** indicate *p* values of <0.05, <0.01 and <0.001, respectively, as determined by unpaired Student´s test. ns, not significant.

To further characterize the DF-1 TLR3 KO cells, we performed luciferase reporter assays to analyze the activation of the IFN-β and NF-κB promoters upon treatment with dsRNA in DF-1 TLR3 KO cells in the absence or presence of exogenously expressed *TLR3* gene. For this, DF-1 WT and DF-1 TLR3 KO cells were transfected with either the IFN-β or NF-κB reporter plasmids, together with a single dose of the empty pcDNA3 expression plasmid as control, or with different doses of the pc-chTLR3-His plasmid expressing TLR3 in the case of DF-1 TLR3 KO cells. At 8 h pt cells were transfected with synthetic dsRNA (Poly I:C) and were harvested 16 h later. As shown in **Figures 9A, B**, when DF-1 TLR3 KO cells were transfected with the empty expression plasmid the activation of the IFN-β and NF-κB promoters was significantly lower than that found in DF-1 WT cells. However, the impairment of IFN-β and NF-κB promoter activation caused by TLR3 knockout could be rescued by TLR3 overexpression in a dose-dependent manner, reaching with the highest doses of plasmid activity levels comparable to those obtained in DF-1 WT cells. These results demonstrate that the downregulation of the innate immune response in DF-1 TLR3 KO cells is exclusively due to the absence of TLR3 protein.

In a similar set of experiments, cells were then either treated with the synthetic dsRNA Poly I:C (20 μg) added directly to the culture medium or transfected with different amounts of Poly I:C (100, 200 or 300 ng) at 8 h pt with the IFN-β and NF-κB reporter plasmids. 16 h later cells were harvested, and luciferase activity quantified. As shown in **Figure 9**, while addition of Poly I:C readily activated IFN-β (**Figure 9C**) and NF-κB (**Figure 9D**) promoters in DF-1 WT cells, DF-1 TLR3 KO cells did not respond to the same treatment. Furthermore, as shown before, transfection of Poly I:C into DF-1 WT cells produced a significant dose-dependent increase in IFN-β promoter activity. Again, this treatment induced some activation of the IFN-β in DF-1 TLR3 KO cells, but the highest activity attained in these cells was significantly lower (40-50%) than that in DF-1 WT cells (**Figure 9C**). Similarly, lower levels of NF-κB promoter activation were detected in DF-1 TLR3 KO transfected with Poly I:C in comparison with DF-1 WT cells (**Figure 9D**).

To assess the contribution of the cytoplasmic sensor MDA5 to the activation of the IFN-β promoter by Poly I:C, a similar experiment was conducted with sets of cells transfected with the *MDA5-*specific or control siRNA, and subsequently treated or transfected with Poly I:C. The silencing of *MDA5* in DF-1 WT cells resulted in a slight reduction in IFN-β promoter activity in cells treated with Poly I:C. As in the previous experiment, only residual activity could be detected in DF-1 TLR3 KO cells, regardless of the treatment with the *MDA5*-specific siRNA or the control siRNA (**Figure 9E**). However, in *MDA5*-silenced DF-1 WT cells transfected with each of the three doses of Poly I:C there was a notable decline in IFN-β promoter activity in comparison with cells transfected with the control siRNA. This reduction was comparable in magnitude to that observed in DF-1 TLR3 KO cells, which was even more pronounced when these cells were transfected with the *MDA5*-specific siRNA. Overall, these results demonstrate that endosomal stimulation by Poly I:C induces mainly a TLR3-dependent activation of IFN-β and NF-κB promoters. Conversely, cytoplasmic delivery of Poly I:C by transfection activated both MDA5- and TLR3-signaling pathways. Activation of the TLR3-dependent pathway by transfected Poly I:C suggests that the dsRNA was captured by endosomes in the cytoplasm.

To further confirm the role of TLR3 in the detection of transfected Poly I:C, we used BFA to prevent endosomal acidification, which is required for TLR activation (Hacker et al., 1998; Yi et al., 1998). Therefore, DF-1 WT and DF-1 TLR3 KO previously transfected with the IFN-β reporter plasmid were transfected with Poly I:C (250 ng) and subsequently treated with BFA (50 μM) or the vehicle dimethyl sulfoxide (DMSO) 1 h later. Following a 16 h incubation period, the cells were harvested, and the luciferase activity quantified. **Figure 9F** shows that treatment with BFA of Poly I:C-transfected DF-1 WT cells resulted in a notable reduction (42%) in IFN-β promoter activity, approaching the result obtained in DF-1 TLR3 KO cells in the absence of BFA. Significantly, a reduction in IFN-β promoter activity was also observed upon treatment of DF-1 TLR3 KO cells with BFA, indicating that in addition to TLR3, other TLRs could also be activated in IBDV-infected cells.

### Endosomal acidification is required for TLR3-mediated apoptosis induction in IBDV-infected DF-1 cells

**Figure 10:**
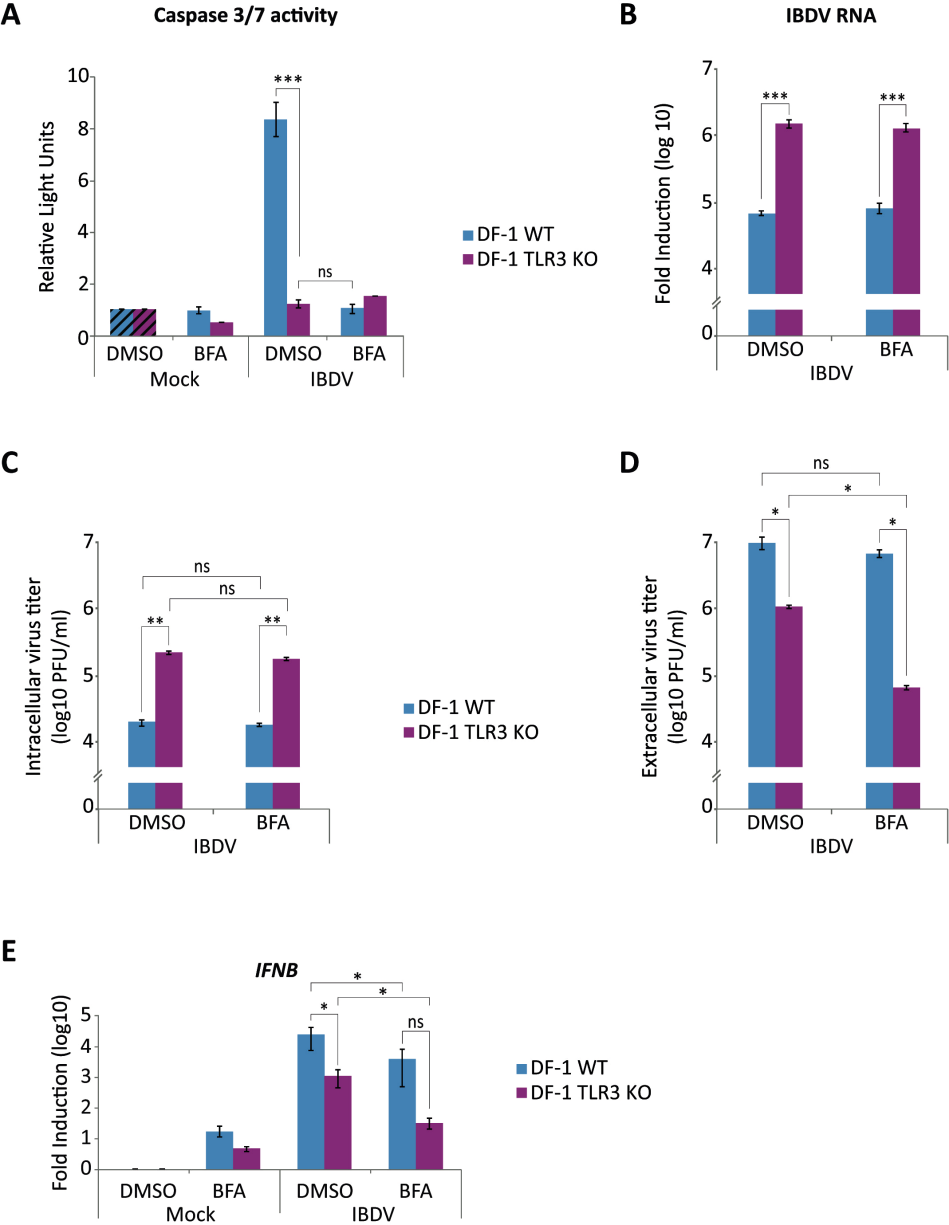
Inhibition of endosomal acidification by treatment of IBDV-infected DF-1 cells with BFA abrogates apoptotic cell death. DF-1 and DF-1 TLR3 KO cells mock-infected or infected with IBDV (MOI of 2 PFU/ml) were treated or not with BFA (50 µM) after 2 h pi. **(A)** Apoptosis was measured by using the Caspase-Glo 3/7 assay kit, and each determination was carried out in duplicate. Caspase values from infected cell samples were normalized to those from mock-infected cells (Mock) (Striped bars). **(B)** The accumulation of IBDV RNA was determined by SYBR green-based RT-qPCR. Analysis of extracellular **(C)** and intracellular **(D)** virus titers. **(E)** RT-qPCR study of the expression of *IFNB* gene. Cellular gene expression values were normalized to those of the *GAPDH* mRNA content and are presented on a log_10_ scale as the fold induction over the level found in mock-infected DF-1 or DF-1 TLR3 KO cells not treated with BFA. Bars indicate means ± standard deviations based on data of duplicate samples from three independent experiments. *, ** and *** indicate *p* values of <0.05, <0.01 and <0.001, respectively, as determined by unpaired Student´s test. ns, not significant.

Given that endosomal acidification is a prerequisite for TLR3-dependent signaling, we sought to ascertain the impact of the BFA treatment on the induction of apoptosis in response to IBDV infection. Therefore, DF-1 WT and DF-1 TLR3 KO cells infected with IBDV were treated with BFA or the vehicle DMSO 2 h later and caspase 3/7 activity was analyzed at 24 h pi. As observed in previous experiments, a significant reduction in luciferase levels was evident in DF-1 TLR3 KO cells in comparison with DF-1 WT cells. It is noteworthy that treatment of DF-1 WT cells with BFA resulted in a marked reduction in caspase 3/7 activity, with luciferase values comparable to those observed in DF-1 TLR3 KO cells (**Figure 10A**). These findings further substantiate the pivotal role of TLR3 in apoptosis induction by IBDV infection. To confirm that BFA does not affect IBDV replication under the conditions used, we conducted an RT-qPCR analysis to quantify the accumulation of viral RNA in samples from cells treated as described above. As illustrated in **Figure 10B**, the RNA accumulation values were markedly higher in DF-1 TLR3 KO cells compared to DF-1 WT cells, regardless of BFA treatment. Neither BFA treatment affected the accumulation of IBDV RNA in DF-1 WT cells. We also analyzed the production of intra- and extracellular infectious virus by virus titration from both, cell supernatants and extracts from infected cell cultures. As shown previously, the accumulation of intracellular virus was higher in DF-1 TLR3 KO cells, and BFA treatment did not alter the rate of virus accumulation in either cell line (**Figure 10C**). On the other hand, the titer of extracellular virus was again significantly higher in DF-1 WT cells compared to DF-1 TLR3 KO cells, and BFA treatment produce a slight, not significant, reduction in virus titer in DF-1 WT cells. A more pronounced effect was observed in BFA-treated DF-1 TLR3 KO cells (**Figure 10D**). A possible explanation for the reduction in extracellular virus titers could be the presence of BFA in the supernatants of BFA-treated cells, which could interfere with endocytic virus entry into the cells of the cultured monolayers used for titration. Indeed, pretreatment of DF-1 cells with BFA for 30 min before infection causes a five to ten-fold reduction in both extracellular and intracellular virus yields as determined at 24 h pi (data not shown).

*IFNB* gene expression was also analyzed in samples from BFA treated DF-1 WT and DF-1 TLR3 KO cells (**Figure 10E**). In BFA-untreated samples we observed a diminished expression in DF-1 TLR3 KO cells as compared to DF-1 WT cells, as previously shown. An additional reduction was observed in both DF-1 WT and DF-1 TLR3 KO cells treated with BFA. The decrease of *IFNB* gene expression in BFA-treated DF-1 TLR3 KO could again indicate that other TLR can also be activated in IBDV-infected cells. Although the effect of BFA treatment on *IFNB* gene expression in DF-1 WT cells was clear and statistically significant, it was not as drastic as the effect on apoptosis (compare **Figures 10A, E**). This result suggests that TLR3 deficiency has a direct effect on apoptosis, in addition to its indirect effect through the reduction of *IFNB* gene expression.

### Role of RIPK1 in IBDV-mediated apoptosis triggering

**Figure 11:**
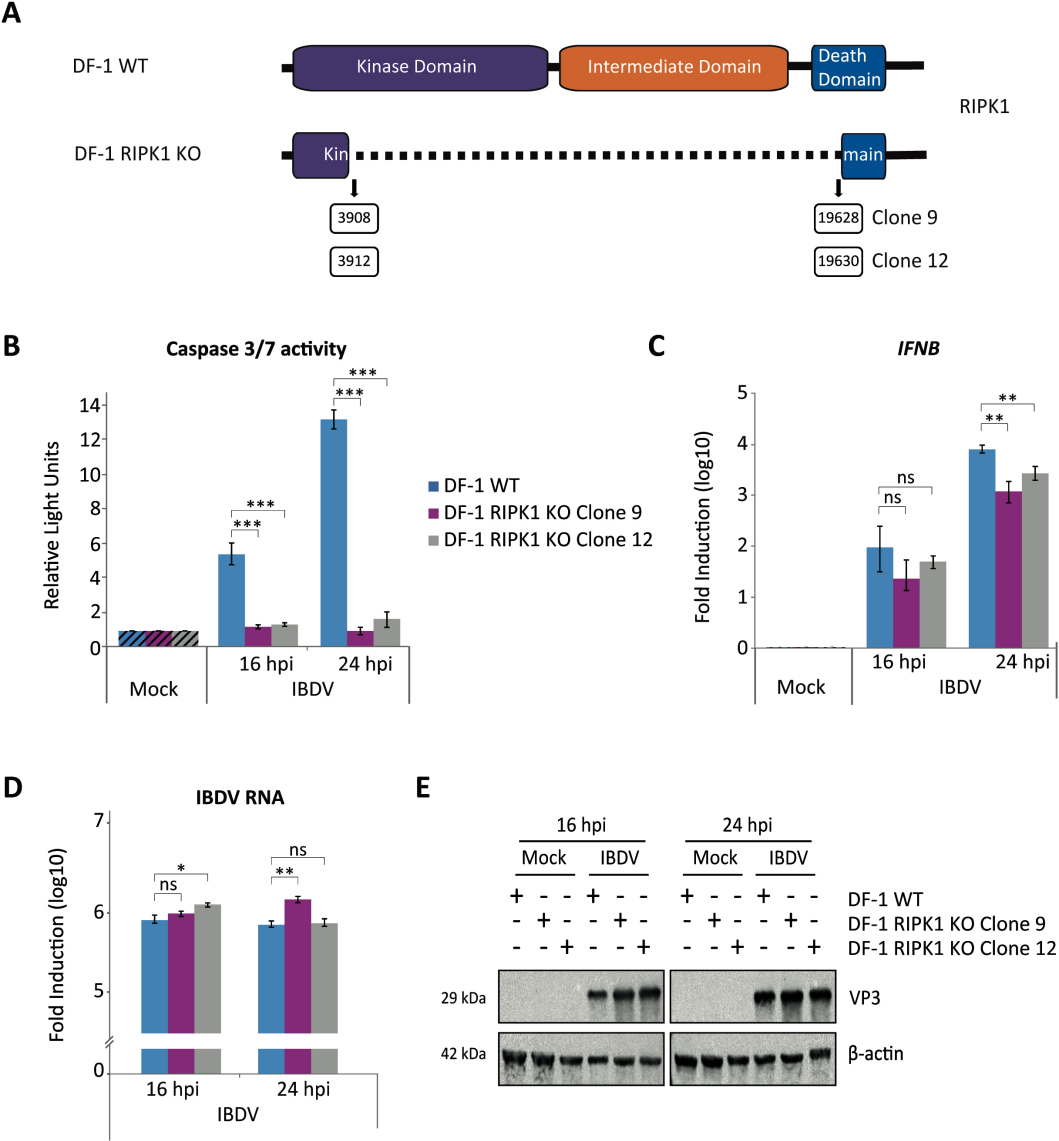
Ablation of RIPK1 expression abrogates IBDV-mediated apoptosis triggering. **(A)** Schematic representation of the structure of the *RIPK1* gene and the deleted versions of the gene present in two selected DF-1 RIPK1 KO cell clones. The boxed numbers below the scheme indicate the nucleotide positions at which the deletions occurred in each cell clone. **(B-E)** DF-1 and DF-1 RIPK1 KO cells from clones 9 and 12 were mock-infected or infected with IBDV (MOI of 2 PFU/cell) and cells were harvested at 16 and 24 h pi. **(B)** Apoptosis was measured by using the Caspase-Glo 3/7 assay kit, and each determination was carried out in duplicate. Caspase values from infected cell samples were normalized to those from mock-infected cells (Mock) (Striped bars). Bars indicate means ± standard deviations based on data of duplicate samples from three independent experiments. **(C, D)** The expression levels of the *IFNB* gene **(C)** and the accumulation of viral RNA **(D)** were analyzed by SYBR green-based RT-qPCR. Recorded values for *IFNB* gene were normalized to the *GAPDH* mRNA content and are presented on a log_10_ scale as the fold induction over the level found in mock-infected DF-1 or DF-1 RIPK1 KO cells. Bars indicate means ± standard deviations based on data of duplicate samples from three independent experiments. **(E)** Western blot analysis of total cell extracts with antibodies specifically recognizing the IBDV structural VP3 protein. Antibodies to β-actin were used for protein loading control. *, ** and *** indicate *p* values of <0.05, <0.01 and <0.001, respectively, as determined by unpaired Student´s test. ns, not significant.

About two decades ago, several laboratories described that TLR3 can directly trigger caspase-8-dependent apoptosis in human cancer cells upon activation by dsRNA, a process that requires the formation of a complex involving caspase 8, TLR3 and TRIF (Salaun et al., 2006, 2007). Later, it was demonstrated that recruitment and activation of caspase 8 to TLR3 requires the participation of RIPK1 (Estornes et al., 2012). In view of our previous results suggesting a direct role of TLR3 in apoptosis triggering in IBDV-infected cells, additionally to its contribution through IFN-β production, we sought to investigate the potential involvement of RIPK1 in this apoptotic process. After multiple unsuccessful attempts to significantly knock down RIPK1 expression using siRNA, we employed CRISPR/Cas9 gene editing to generate a large deletion (~15.7 kb) in the *RIPK1* gene of DF-1 cells, spanning from exon 2 to exon 12, the last exon of the gene (**Figure 11A**). Two clones, designated 9 and 12, displaying minor variations in deletion length, were selected for further analysis from the pool of genetically characterized clones. First, we evaluate the fate of RIPK1 deficient DF-1 cells following IBDV infection. For this, DF-1 WT and DF-1 RIPK1 KO cells from both clones were infected and samples were taken at 16 and 24 h pi for analysis of caspase 3/7 activity. Strikingly, a marked reduction in apoptosis levels was observed in infected cells from both RIPK1 KO cell lines compared to DF-1 WT cells at both time points pi (**Figure 11B**). This effect was particularly pronounced at 24 h pi, when apoptosis levels in DF-1 WT cells were notably higher. Remarkably, as it occurred with DF-1 TLR3 KO cells, the extent of apoptosis in both cell clones following IBDV infection was comparable to that measured in uninfected control cells. With respect to DF-1 WT cells, the expression of *IFNB* gene in the absence of RIPK1 was also reduced at both times pi for each of the two clones analyzed (**Figure 11C**), although the differences were only statistically significant at 24 h pi. Moreover, the reduction of *IFNB* gene expression was below 1-log unit, which is lower than that observed in the DF-1 TLR3 KO cells (compare **Figures 8A**, **11C**). Accordingly, only minor differences in the accumulation of viral RNA between DF-1 WT and DF-1 RIPK1 KO cells were observed at the two times pi analyzed (**Figure 11D**). Nonetheless, an increase in VP3 protein expression is also observed, particularly at 16 h pi (**Figure 11E**). Overall, these findings indicate that RIPK1 is a key mediator of apoptosis induction in DF-1 cells following IBDV infection, while also contributing partially to IFN-β signaling, as evidenced by the modest reduction in *IFNB* gene expression observed in RIPK1-deficient cells.

### TLR3 deletion does not affect replication of ARV, VSV, SFV and NDV

**Figure 12:**
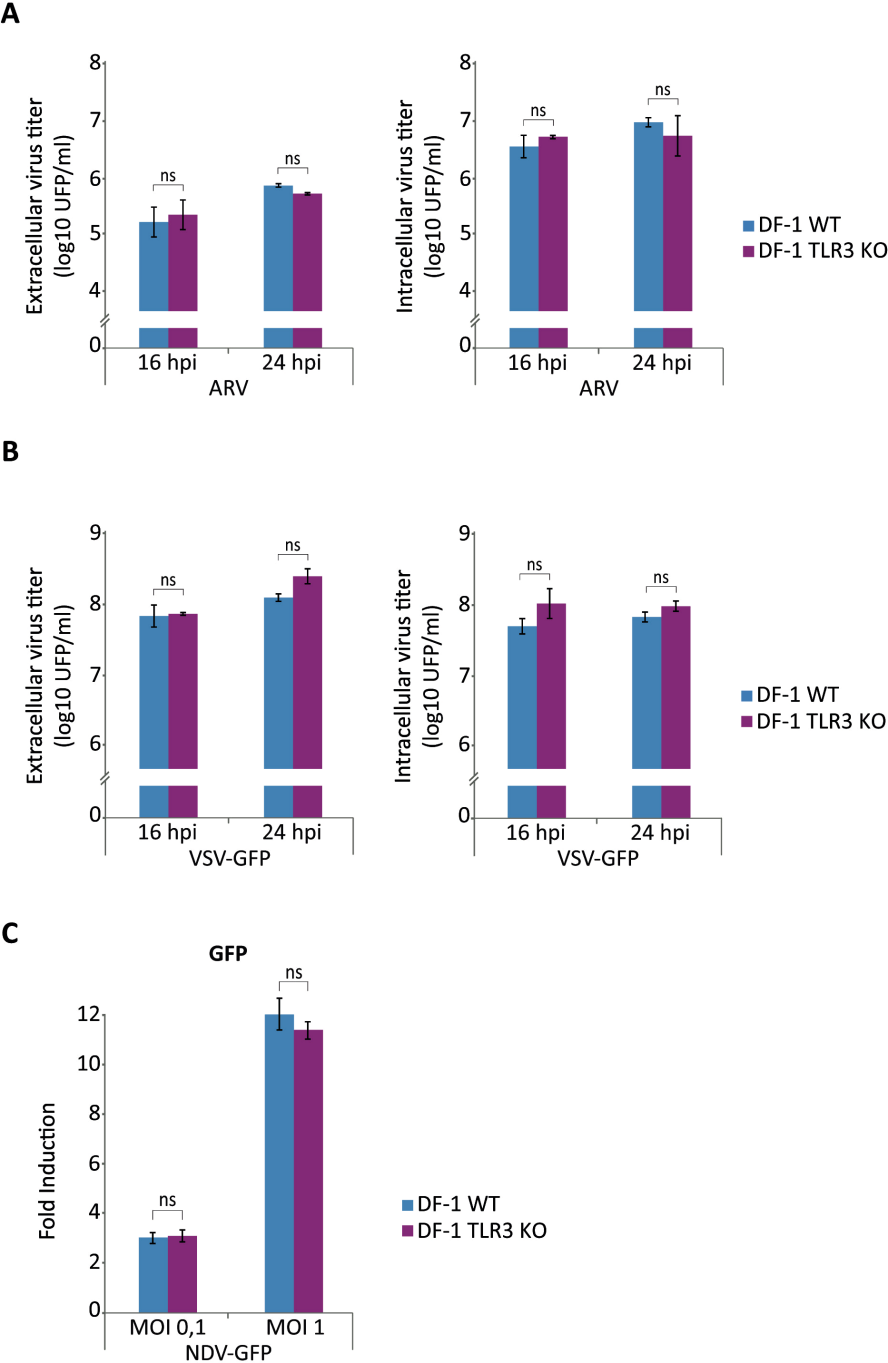
TLR3 deletion does not influence ARV, VSV-GFP and NDV-GFP replication. **(A, B)** DF-1 and DF-1 TLR3 KO cells were mock-infected or infected with ARV **(A)** and VSV-GFP **(B)** (MOI of 2 PFU/cell) and cells were harvested at 16 and 24 h pi for analysis of extracellular and intracellular virus titers. **(C)** DF-1 and DF-1 TLR3 KO cells were mock-infected or infected with NDV-GFP at MOIs of 0.1 and 1 PFU/cell and cells were harvested at 24 h pi for analysis of GFP expression. Recorded values for GFP fluorescence were normalized to those found in mock-infected DF-1 or DF-1 RIPK1 KO cells. Bars indicate means ± standard derivations based on duplicate samples from three independent experiments. ns, not significant.

The enhancement of IBDV replication and its intracellular accumulation in cells that do not express TLR3 is consistent with data supporting the involvement of TLR3 in the regulation of the innate immune response and in the activation of apoptosis induced by IBDV infection. We therefore decided to investigate whether this effect is specific to IBDV infection or if the absence of TLR3 also affects the replication of other RNA viruses. To this end, DF-1 WT and DF-1 TLR3 KO cells were infected with ARV (another dsRNA virus), VSV-GFP (a negative polarity ssRNA virus expressing GFP), and SFV (a positive polarity ssRNA virus) at an MOI of 2 PFU/cell, and the extra- and intracellular viral titers quantified at 16 and 24 h pi. Both intra- and extracellular titers of ARV (**Figure 12A**), VSV-GFP (**Figure 12B**) and SFV (data not shown) did not vary depending on the infected cell line, DF-1 WT or DF-1 TLR3 KO. Additionally, we assessed NDV-GFP replication in both DF-1 WT and DF-1 TLR3 KO cell lines by quantifying GFP fluorescence. Cells were infected at MOIs of 0.1 and 1 PFU/cell, and samples were collected at 24 h pi. No significant differences in GFP expression were observed between the two cell lines at either MOI tested (**Figure 12C**).

## Discussion

IBDV poses a significant challenge to the host immune defenses of juvenile chickens, as it targets and eliminates developing B lymphocytes within the bursa of Fabricius, which is the primary lymphoid organ responsible for B cell maturation and the development of a diverse antibody repertoire in birds. Extensive B lymphocyte depletion due to apoptotic cell death causes an irreversible atrophy of the organ, resulting in profound and long-lasting immunosuppression (Sharma et al., 2000). Various studies have provided evidence implicating the viral proteins VP2 (Fernández-Arias et al., 1997; Qin et al., 2017) and VP5 (Yao and Vakharia, 2001; Li et al., 2012) in the apoptotic process. Conversely, our previous results demonstrated that exposure to type I IFN in IBDV-infected cells induces extensive cell death through apoptosis, indicating that IFN could be pivotal in the pathogenesis associated with this virus.

In this study, we extended these observations and demonstrated that treatment of chicken fibroblasts, both from primary CEF cultures or from the immortalized DF-1 cell line, with chIFN-α shortly after IBDV infection significantly anticipates the onset of CPE and caspase-3/7 activation compared to untreated cultures. Consistently higher levels of caspase-3/7 activity were observed in CEF than in DF-1 cells under all conditions. This is in line with previous reports indicating elevated SOCS1 expression and downregulation of apoptosis-related genes in DF-1 cells, which may attenuate their responsiveness to IFN and death signals (Kong et al., 2011; Giotis et al., 2017).

Conducting the experiments described above in primary B lymphocytes would have further strengthened our conclusions. In this regard, recent studies have demonstrated the feasibility of performing IBDV infections in *ex vivo* cultures of B lymphocytes isolated from the bursa of Fabricius (Dulwich et al., 2018; Soubies et al., 2018). Nevertheless, these cells undergo rapid spontaneous apoptosis under ex vivo conditions unless the culture medium is supplemented with survival factors such as CD40L, which supports B-cell viability, proliferation, and functional maintenance (Kothlow et al., 2008). Importantly, CD40L signaling also activates anti-apoptotic pathways, and its inclusion may consequently interfere with the accurate evaluation of IBDV- and/or IFN-induced apoptotic responses. Nonetheless, we confirmed that a similar effect of IFN to that observed in CEF and DF-1 cells was attained in the DT40 B-cell line, suggesting that the signaling mechanisms driving the apoptotic response are also active in B lymphocytes.

The pro-apoptotic contribution of endogenous IFN signaling was demonstrated by the pharmacological blockade of the JAK/STAT pathway using Ruxolitinib (Rx). In DF-1 cells, Rx treatment strongly reduced the expression of the *IFNB*, *MDA5*, *TLR3* and *Mx* genes, indicating a broad suppression of the innate response. At the same time, it almost completely prevented caspase-3/7 activation at late times pi. In parallel, inhibition of viral replication led to a drastic drop in caspase activity. Overall, these results suggest that IBDV dsRNA is the critical viral trigger for apoptosis (Cubas-Gaona et al., 2018), and that type I IFN, produced downstream of dsRNA sensing, amplifies this apoptotic program rather than merely establishing an antiviral state. Previous studies have shown that recognition of IBDV genomic dsRNA by MDA5 activates the MAVS-dependent signaling cascade to induce type I IFN expression (Lee et al., 2014; Ye et al., 2014; Lee et al., 2015b; Diaz-Beneitez et al., 2022). Consistently, our results show that the silencing of *MDA5* and *MAVS* genes individually or in combination in DF-1 cells prior to infection attenuated *IFNB* gene expression, and resulted in a significant, yet partial, reduction in apoptosis induction, allowing enhanced IBDV replication. Thus, while these cytosolic sensors clearly contribute to both antiviral signaling and cell death, their abrogation does not fully resolve either phenotype, suggesting that additional pathways must be involved.

Both type I and type II IFNs are known to restrict IBDV replication *in vitro* and *in vivo* (Mo et al., 2001; Song et al., 2017; Cubas-Gaona et al., 2018). However, our findings are consistent with previous studies that link type I IFN responses with the severity of IBDV-induced pathology. Pathogenic and very virulent IBDV strains have been shown to induce stronger or dysregulated IFN responses, which correlate with increased bursal damage and mortality (Gelb et al., 1979; Eldaghayes et al., 2006; Aricibasi et al., 2010). Furthermore, it has been reported that susceptible chicken lines mount earlier and stronger IFN responses than more resistant breeds (Aricibasi et al., 2010). Together, these studies emphasize that, while IFNs are crucial for antiviral defense, excessive or premature activation can exacerbate immunopathology and promote disease progression. The dual context-dependent role of type I IFNs has been well documented in various viral infection models, including human immunodeficiency virus (HIV), lymphocytic choriomeningitis virus (LCMV), and severe acute respiratory syndrome coronavirus (SARS-CoV), in which systemic IFN responses contribute to either immune suppression or enhanced pathology (Wilson et al., 2013; Anjum et al., 2021; Teer et al., 2025).

The ability of the JAK/STAT pathway inhibitor Rx to suppress apoptosis so effectively *in vitro*, together with previous reports demonstrating the feasibility of its *in vivo* administration (Elli et al., 2019), suggests that this compound could be useful for investigating the contribution of IFN signaling to IBDV-induced pathology in animal models.

### PKR contributes to apoptosis in IBDV-infected cells, although it is not the primary driver of this process

PKR is an ISG that acts as a sensor of dsRNA and plays a key role in regulating antiviral responses and apoptosis (McAllister and Samuel, 2009; Lee et al., 1997; Lemaire et al., 2005). It has also emerged as an important player in our system. Our previous studies in HeLa cells have shown that PKR is pivotal for apoptosis induced by IBDV dsRNA in the presence of IFN-α (Cubas-Gaona et al., 2018). Furthermore, the upregulation of PKR and other ISGs has been reported in both IBDV-infected cell cultures (Lee et al., 2015a; Ouyang et al., 2017) and the bursa and spleen of infected chickens (Smith et al., 2015). Then, to investigate the potential role of PKR in IBDV-induced apoptosis in chicken cells we generated DF-1 PKR KO cells. In these cells, we observed a substantial protection from virus-induced CPE and reduced *IFNB* expression, consistent with the requirement of PKR for the full induction of type I IFN, as described for its mammalian counterpart (McAllister and Samuel, 2009; Pham et al., 2016). However, apoptosis was not completely abolished in PKR-deficient cells, suggesting that, while PKR contributes to IBDV-induced cell death, it is not the primary executor in this avian context.

### TLR3 plays a crucial role in IFN expression and apoptosis in IBDV-infected DF-1 cells

Since neither MDA5/MAVS silencing nor PKR deletion completely prevented the induction of IFN-β or the triggering of apoptosis, our attention turned to the endosomal receptor TLR3. Several studies have suggested that TLR3 expression increases during IBDV infection both *in vitro* and *in vivo*, reinforcing its proposed role in the innate immune response to the virus (Smith et al., 2015; He et al., 2017; Kumar et al., 2010). Nevertheless, the direct involvement of TLR3 in IFN-β induction in IBDV-infected cells remains to be confirmed.

To explore the role of TLR3 during IBDV infection we generated DF-1 TLR3 KO cell lines using the CRISPR/Cas9 technology. Microscopic examination of IBDV-infected TLR3 KO cultures revealed a striking absence of CPE at late times pi, with caspase-3/7 activity remaining at basal levels, comparable to those observed in mock-infected controls. At the transcriptional level, these cells exhibited a significant decrease in *IFNB*, *MDA5, Mx* and *OAS* gene expression after infection, pointing to TLR3 as a central mediator of both antiviral signaling and apoptosis in chicken fibroblasts. Consistent with a cooperative model of innate sensing, knocking down *MDA5* in TLR3-deficient cells caused an additional decrease in IFN-β induction. This suggests that TLR3 provides the primary input while MDA5 amplifies or sustains the response.

Transactivation reporter assays further underscored the pivotal role of chTLR3 in the activating the IFN-β and NF-κB promoters in response to dsRNA stimulation. The impaired activation of both reporter elements in TLR3-deficient cells following Poly I:C transfection was restored in a dose-dependent manner by expressing chTLR3 from a plasmid vector. This experimental system enabled us to assess the relative contributions of TLR3 and MDA5 to the transactivation of the IFN-β and NF-κB promoters when cells were exposed to Poly I:C, either via extracellular (endosomal) treatment or intracellular (cytoplasmic) transfection. While DF-1 WT cells responded to stimulation by either route, the addition of Poly I:C to the culture medium failed to activate both promoters in TLR3 KO cells. However, *MDA5* silencing had only a minor impact in IFN-β promoter activation in DF-1 WT cells treated with Poly I:C. By contrast, when Poly I:C was delivered by transfection, both *TLR3* deletion and *MDA5* knockdown reduced IFN-β promoter activity independently, with a more pronounced decrease observed upon combined inhibition of both PRRs. These findings indicate that extracellular Poly I:C primarily activates TLR3-dependent signaling pathways, which is consistent with its endocytic uptake, whereas cytoplasmic delivery triggers both MDA5- and TLR3-mediated responses. The fact that transfected Poly I:C can also trigger TLR3 signaling suggests that cytoplasmic dsRNA may be entrapped in endosomes, a mechanism that has been reported previously in HeLa cells microinjected with Poly I:C (Zuo et al., 2022). In line with this, inhibiting endosomal acidification by treating Poly I:C transfected DF-1 WT cells with BFA significantly reduces IFN-β promoter activity. Interestingly, we also observed a clear, albeit non-significant, reduction in IFN-β promoter activity in DF-1 TLR3 KO cells following BFA treatment. This could suggest that Poly I:C transfection may also activate other endosomal receptors, such as TLR7, whose expression is also upregulated after IBDV infection (Rauf et al., 2011; Smith et al., 2015).

Notably, inhibiting endosomal acidification during IBDV infection of DF-1 WT cells by treatment of DF-1 WT cells with BFA after virus adsorption completely abrogated apoptosis, while only partially reducing *IFNB* transcription, closely mimicking the phenotype of TLR3 KO cells. A minor effect on *IFNB* gene expression was also observed in DF-1 TLR3 KO cells, again suggesting the potential activation of TLR7. Importantly, this treatment did not impair intracellular viral RNA accumulation or overall virus production, ruling out the possibility that the reduction in apoptosis was simply due to infection being inhibited. Only a slight decrease in extracellular titers from BFA-treated TLR3 KO cells was detected, which may be attributable to residual drug interfering with virus endocytic uptake during titration. Consistent with this, pretreating of DF-1 cells with BFA prior to infection resulted in a five- to tenfold decrease in both extracellular and intracellular virus yields at 24 h pi. Together, these results suggest that, rather than being strictly coupled to IFN-β induction, TLR3-mediated cell death is also due to a direct, IFN-independent contribution of TLR3 to apoptosis regulation.

### Role of TLR3 in the control of viral replication and spread

DF-1 TLR3 KO cells accumulated higher amounts of VP3 protein and viral RNA than WT cells, yet they yielded lower extracellular titers. These findings suggest that TLR3 deficiency favors intracellular viral replication but compromises viral release. One possible explanation is that the massive apoptosis observed in WT cultures facilitates virus shedding, whereas its absence in TLR3 KO cells limits dissemination. However, our previous data show that reduced apoptosis usually correlates with higher extracellular titers, as seen for other RNA viruses such as Sendai virus (SeV) and Sindbis virus (SINV) (Zuo et al., 2022). An alternative explanation is that TLR3 modulates host pathways directly involved in viral egress. However, this possibility remains purely speculative at present.

No significant differences in viral titers were observed between DF-1 WT and TLR3 KO cells when testing other RNA viruses (ARV, VSV, SFV and NDV) that enter cells via endocytosis. These results are consistent with previous findings in TLR3-deficient quail fibroblasts, in which the loss of TLR3 impaired the induction of IFN-β by Poly(I:C), but did not affect the replication of ARV, VSV or IAV (Mahesh et al., 2020). In DF-1 cells, MDA5 is the primary RNA sensor for IAV and Poly(I:C), with TLR3 playing a secondary role (Lee et al., 2020). More recently, Lee et al. (2023) showed that TLR3 deletion in DF-1 cells slightly enhances NDV replication, with stronger effects upon dual *MDA5/TLR3* knockout, though without clear correlation to IFN expression. In light of these findings, we infected both DF-1 WT and DF-1 TLR3 KO cells with a recombinant NDV-GFP virus and found comparable levels of GFP expression in both cell types, suggesting that TLR3 does not play a major role in restricting NDV replication under our experimental conditions. Together, these results suggest that TLR3 is not involved in triggering innate immune response upon infection with ARV, VSV, SFV and NDV in DF-1 cells. While the ssRNA genomes of VSV, SFV, and NDV are not canonical ligands for TLR3, avian reovirus (ARV), like IBDV, possesses a dsRNA genome that could in principle activate TLR3. However, unlike IBDV, the ARV genome remains enclosed within the viral core particle and is therefore largely shielded from endosomal TLR3 recognition. Interestingly, Lee et al. (2023) also reported Poly(I:C)-induced cytopathic effects in DF-1 WT and MDA5 KO cells, but not in TLR3 KO cells. Although not confirmed as apoptosis, these results align with our evidence that chicken TLR3 mediates apoptosis in response to viral or synthetic dsRNA. To our knowledge, this study provides the most compelling evidence to date of the antiviral function of chicken TLR3 during IBDV infection, as well as its critical involvement in the induction of apoptosis. These findings highlight the dual role of TLR3 in orchestrating both innate immune responses and regulating virus-induced cell death.

### Role of RIPK1 downstream of TLR3 in the induction of apoptosis in IBDV-infected DF-1 cells

In order to analyze the downstream mechanisms by which TLR3 promotes apoptosis, we examined the function of RIPK1, a protein adaptor that has previously been associated with TLR3- and TRIF-mediated apoptotic signaling in mammalian cells (Bianchi et al., 2017). To this end, DF-1 RIPK1 KO cells were generated. Upon infection with IBDV, DF-1 RIPK1 KO cells exhibited a dramatic reduction in caspase-3/7 activation, reaching levels similar to or only slightly higher than those observed in mock-infected cultures. Meanwhile, *IFNB* gene induction was only modestly reduced, and, consequently, only a slight increase in viral RNA accumulation was observed in DF-1 RIPK1 KO cells. These data suggest that RIPK1 is crucial for TLR3-driven apoptosis in chicken fibroblasts, yet its impact on IFN-β expression is minimal, supporting the notion that the apoptotic function of TLR3 is partially independent of its role in canonical antiviral signaling. To our knowledge, these results provide the first experimental evidence of the essential role of RIPK1 in virus-induced cell death in avian systems, and highlights the importance of TLR3-RIPK1 signaling in shaping the pathogenesis of IBDV by modulating host cell fate. The presence of functional homologs of most key mammalian components of this pathway in the chicken genome indicates that the signaling cascade is likely conserved across species (Gillespie et al., 2011). Nonetheless, additional research is needed to fully define how the RIPK1/TLR3/TRIF axis leads to caspase activation in chicken cells.

In our previous study using HeLa cells, we demonstrated that IBDV genomic dsRNA plays a pivotal role in inducing apoptosis, with PKR acting as a key mediator of this process. However, the present study in chicken DF-1 cells reveals that while PKR also contributes to apoptosis upon IBDV infection, TLR3 is the primary mediator. Interestingly, the ablation of TLR3 expression alone is sufficient to completely prevent IBDV-induced cell death. Recently, Zuo et al. (2022) described that intracytoplasmic injection or transfection of synthetic dsRNA induces extensive apoptosis in HeLa cells, a process that is further enhanced by type I IFN priming. In contrast to our findings with DF-1 cells, they reported that both PKR and TLR3 are essential for the complete induction of dsRNA-mediated apoptosis in these cells. Taken together, these results suggest that, while dsRNA-triggered apoptotic pathways are conserved across species, the molecular dependencies differ between human and avian systems, likely reflecting evolutionary divergence in the architecture of innate immune signaling networks.

Our findings provide new insights into the mechanism of induction of the innate immune response during IBDV infection and its close relationship with the activation of the apoptotic death process. Based on all the data, we propose a model in which IBDV genomic dsRNA is first unveiled in endosomes after capsid disassembly. This process is facilitated by low calcium and acidic pH, as well as by amphipathic peptides such as pep46, which are associated with the capsid. This dsRNA is then recognized by TLR3, which triggers TRIF–RIPK1 signaling and initiates both IFN-β induction and a potent pro-apoptotic cascade. Following endosomal escape, the viral genome replicates in the cytoplasm, where newly synthesized dsRNA is recognized by MDA5 and PKR, further amplifying IFN-β expression and apoptosis. In parallel, a fraction of cytoplasmic dsRNA may be re-internalized into endosomes, where it can reactivate TLR3 (Zuo et al., 2022). TLR3 thus operates at the intersection of two partially separable outputs: an antiviral arm that promotes IFN production and ISG expression, and a RIPK1-dependent arm that drives extensive apoptosis. Late IFN-β secretion reinforces this death process, contributing to the profound cytopathic effect observed in infected cultures. This study established the first direct link between the TLR3 receptor and activation of the innate response to IBDV, confirming its role as the primary receptor involved in IBDV recognition. Although these results have been obtained entirely *in vitro* using DF-1 fibroblast cells as a model, as mentioned above, different studies have shown that infection with IBDV triggers TLR3 expression in B lymphocytes of the bursa of Fabricius, in intestinal lamina propria cells, and in other tissues of IBDV-infected chickens (Kumar et al., 2010; Rauf et al., 2011; Guo et al., 2012; Smith et al., 2015; Lee et al., 2015a; He et al., 2017; Chen et al., 2022). Moreover, differential regulation of TLR3 expression in bursal B lymphocytes, spleen and gut-associated lymphoid tissue (GALT) of chickens infected with IBDV strains of different virulence has also been reported. A correlation has been observed between TLR3 levels in the bursa of Fabricius and IBDV strain virulence, suggesting that more virulent strains may activate stronger TLR3-mediated responses (Rauf et al., 2011; Smith et al., 2015; He et al., 2017; Eldaghayes et al., 2006; Liu et al., 2010; Chen et al., 2022). Overall, our findings demonstrating that TLR3 signaling pathway plays a significant role in the host immune response to IBDV *in vitro* align with these previous *in vivo* observations, and support a role for TLR3 in IBDV pathogenicity.

Finally, our findings should be viewed in light of the broader, and sometimes contradictory, roles attributed to TLR3 in viral infections and immune-mediated diseases. While TLR3 has been associated with protective immunity against certain RNA and DNA viruses, including poliovirus, coxsackievirus, encephalomyocarditis virus and herpes simplex virus 1 (HSV-1) (Zhang et al., 2013), it has also been linked to excessive inflammation, tissue damage and disease exacerbation in specific viral and inflammatory conditions (Perales-Linares and Navas-Martín, 2013; Hsieh et al., 2025). Our data are more consistent with the latter scenario. While TLR3 clearly contributes to antiviral signaling, it appears to be a key driver of the massive apoptosis that ultimately underlies bursal damage and immunosuppression during IBDV infection. Future *in vivo* studies are required to confirm the extent to which TLR3-dependent pathways, particularly the TLR3–RIPK1 axis, influence disease outcomes in the natural host.

## Data Availability

The original contributions presented in the study are included in the article/**Supplementary Material**. Further inquiries can be directed to the corresponding author.

## Supplementary material

Supplementary Figure 1 TLR3-dependent IBDV-induced cell death can be inhibited by the pan-caspase inhibitor Z-VAD-FMK. DF-1 and DF-1 TLR3 KO cells (clone 10) were mock-infected or infected with IBDV (MOI of 2 PFU/ml) in the presence of the pan-caspase inhibitor Z-VAD-FMK (ZVAD) (50 µM) or DMSO. DF-1 and DF-1 TLR3 KO cells were also treated with Staurosporine (STS) (1 µM) to confirm the ability of DF-1 TLR3 KO to undergo apoptosis in response to other stress inducing stimuli. Cells were harvested at 24 h pi and apoptosis was measured by using the Caspase-Glo 3/7 assay kit in duplicate using samples corresponding to 12,500 cells. Caspase values from infected cell samples were normalized to those from mock-infected cells (Mock) (Striped bars). Bars indicate means ± standard deviations based on data of duplicate samples from three independent experiments. ** indicate *p* values of <0.01, as determined by unpaired Student´s test. ns, not significant.
