# Supplementary figures and images for: Key role of TLR3 in type I IFN expression and apoptosis induction in IBDV-infected chicken fibroblast cells
Source: Front Cell Infect Microbiol. 2026 Mar 6;16:1767950. doi: 10.3389/fcimb.2026.1767950 (PMC13002827; doi:10.3389/fcimb.2026.1767950)

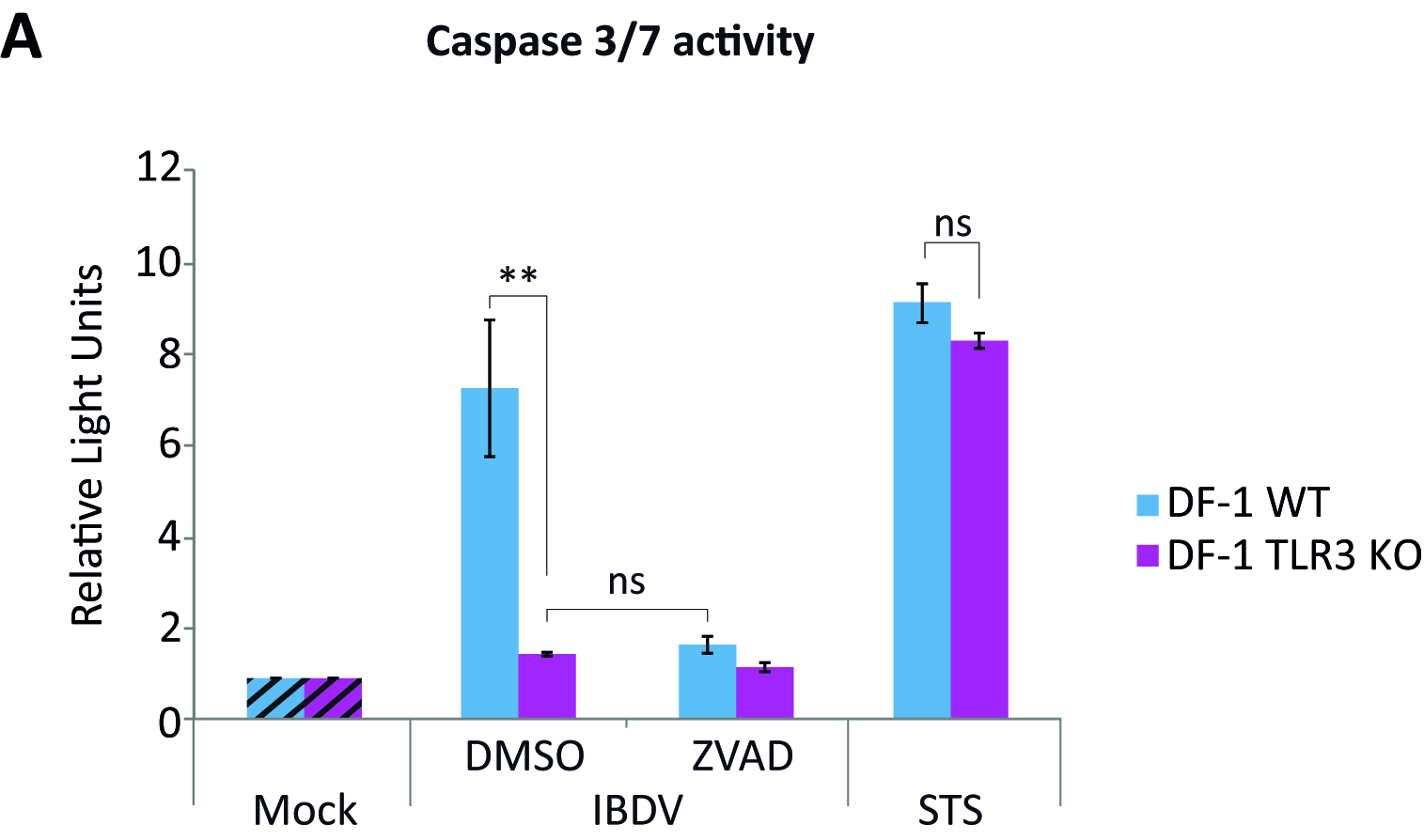

Supplement: Supplementary Figure 1 — TLR3-dependent IBDV-induced cell death can be inhibited by the pan-caspase inhibitor Z-VAD-FMK. DF-1 and DF-1 TLR3 KO cells (clone 10) were mock-infected or infected with IBDV (MOI of 2 PFU/ml) in the presence of the pan-caspase inhibitor Z-VAD-FMK (ZVAD) (50 µM) or DMSO. DF-1 and DF-1 TLR3 KO cells were also treated with Staurosporine (STS) (1 µM) to confirm the ability of DF-1 TLR3 KO to undergo apoptosis in response to other stress inducing stimuli. Cells were harvested at 24 h pi and apoptosis was measured by using the Caspase-Glo 3/7 assay kit in duplicate using samples corresponding to 12,500 cells. Caspase values from infected cell samples were normalized to those from mock-infected cells (Mock) (Striped bars). Bars indicate means ± standard deviations based on data of duplicate samples from three independent experiments. ** indicate p values of <0.01, as determined by unpaired Student´s test. ns, not significant. [file Image1.tif]
